# Intranasal nano-immunotherapy for glioma: bypassing the blood–brain barrier, reprogramming the immune microenvironment, and bridging preclinical validation

**DOI:** 10.3389/fimmu.2026.1950362

**Published:** 2026-10-20

**Authors:** Leina Li, Moli Wu, Liya Li

**Affiliations:** 1 Department of Anesthesiology, Second Affiliated Hospital of Dalian Medical University, Dalian, Liaoning, China; 2 Liaoning Laboratory of Cancer Genomics and Epigenomics, College of Basic Medical Sciences, Dalian Medical University, Dalian, Liaoning, China

**Keywords:** blood–brain barrier, glioma, intranasal delivery, nano-immunotherapy, tumor immune microenvironment

## Abstract

Glioma, particularly glioblastoma, remains difficult to treat because of limited drug penetration across the blood–brain barrier, profound immune suppression, and insufficient intracranial exposure of systemic immunotherapies. Intranasal nano-immunotherapy is being explored as a potential strategy that may integrate noninvasive nose-to-brain delivery with nanocarrier-mediated immune reprogramming. Intranasal administration may partially bypass conventional vascular barriers through olfactory, trigeminal, mucosal, cerebrospinal-fluid, and perivascular transport routes, while nanoparticles are designed to protect immune-active payloads, support brain tumor targeting, and enable controlled release. Current evidence spans three distinct levels: direct intranasal glioma nano-immunotherapy, intranasal glioma nanodelivery without demonstrated immune reprogramming, and non-intranasal glioma nano-immunotherapy that provides mechanistic support for future intranasal adaptation. Across these evidence levels, investigated platforms include peptide-guided nanomicelles, lipid nanoparticles, biomimetic nanoparticles, ferritin-based nanovectors, spherical nucleic acids, extracellular vesicles, bacterial outer membrane vesicles, and stimuli-responsive systems. These platforms can deliver immune-activating cargos, such as STING or TLR agonists; gene-regulatory cargos, such as siRNA and CRISPR-Cas9 components; and microenvironment-modulating cargos, such as chemokine-axis inhibitors, checkpoint-modulating agents, and combination payloads. Through these payloads, intranasal nanoplatforms may activate innate immune sensing, reverse myeloid-mediated suppression, enhance T-cell infiltration, and induce immunogenic cell death. However, translation remains limited by dosing uncertainty, species differences in nasal anatomy, mucosal and neurological safety concerns, manufacturing complexity, and incomplete immune pharmacodynamic validation. This review summarizes recent advances in intranasal and brain-targeted glioma nano-immunotherapy, highlights key immune mechanisms, and proposes a preclinical validation framework for evaluating whether experimental efficacy can be advanced toward clinically testable treatment designs.

## Highlights

- Intranasal nanomedicine may bypass BBB barriers and improve brain-directed glioma therapy.
- Nano-immunotherapy can reprogram myeloid suppression, checkpoints, and T-cell dysfunction.
- Nasal routes, nanoplatforms, and validation criteria are integrated into one framework.

## 1 Introduction

Gliomas, particularly glioblastoma (GBM), remain among the most aggressive and treatment-refractory tumors of the central nervous system, with limited durable benefit despite maximal surgical resection followed by radiotherapy and temozolomide-based chemotherapy (1). Recurrence remains a major clinical challenge because malignant cells can extend beyond the radiographically defined tumor core and persist within infiltrative margins that are difficult to eradicate surgically or pharmacologically. Recent spatial analyses have demonstrated distinct malignant-cell states and tumor-associated programs at the invasive margin, supporting the contribution of residual infiltrative disease to therapeutic failure and recurrence (2, 3). Therapeutic failure in GBM therefore reflects several interconnected barriers, including tumor-cell plasticity and resistance, inadequate intracranial drug delivery, and immune escape within a spatially heterogeneous brain tumor ecosystem (4, 5). A central delivery challenge is the blood–brain barrier (BBB) and the blood–brain tumor barrier (BTB). Importantly, barrier disruption within GBM is neither uniform nor complete. Recent patient-based pharmacokinetic analyses have demonstrated substantial inter- and intratumoral heterogeneity in BBB permeability and drug distribution, including marked differences between contrast-enhancing and non-enhancing tumor regions (6). Whole-brain vascular mapping has likewise revealed spatially heterogeneous vascular remodeling and BBB disruption during glioma progression (7). Consequently, increased permeability within the tumor core should not be assumed to indicate unrestricted access to infiltrative tumor regions. This heterogeneity can limit the penetration of antibodies, nucleic acids, immune agonists, and nanomedicines into therapeutically relevant tumor niches (8). Systemic administration may increase circulating exposure but does not necessarily produce sufficient or selective accumulation within the glioma microenvironment and may increase peripheral exposure. Thus, immune-modulating agents may fail not because their molecular targets are irrelevant, but because adequate exposure and target engagement are not achieved within the appropriate intracranial tumor or immune-cell compartments.

The second major challenge is the immunosuppressive nature of the glioma microenvironment. GBM immune suppression can be organized into three major features: limited effector-cell entry, including poor CD8+ T-cell infiltration and dendritic-cell activation; dominant myeloid suppression, involving tumor-associated macrophages, microglia, and myeloid-derived suppressive programs; and functional immune inhibition, driven by checkpoint signaling and metabolic constraints (9, 10). These features help explain why immune checkpoint blockade, which has transformed the treatment of several extracranial tumors, has shown limited efficacy in unselected GBM populations (11). Recent nano-immunotherapeutic studies have therefore shifted attention from simply killing tumor cells to actively remodeling the glioma immune microenvironment. For example, biomimetic nanoparticles have been used to remodel the immunosuppressive GBM microenvironment and improve antitumor immune responses, while CXCR4/CXCL12-targeted nanoparticles have been designed to interfere with chemokine-mediated immune suppression in glioma (12, 13). The third challenge is that systemically delivered immunotherapies may have an unfavorable therapeutic index in brain tumors. Antibodies, cytokines, nucleic acid therapeutics, STING/TLR agonists, and gene-editing systems can trigger systemic inflammation, immune-related toxicity, or rapid clearance before achieving meaningful intracranial exposure (1, 14). These limitations have motivated the development of local or brain-targeted delivery strategies (15). Intranasal nano-immunotherapy is attractive in this context because it may combine three advantages: noninvasive access to the brain, nanocarrier-mediated protection and targeting of therapeutic payloads, and localized immune reprogramming within glioma-associated niches (16–18). Thus, intranasal nano-immunotherapy may be viewed as a proposed integrated strategy that aims to address delivery barriers and immunological resistance simultaneously, although its clinical relevance remains to be established.

Intranasal delivery has emerged as a promising strategy for brain-targeted therapy because the nasal cavity provides anatomical connections to the central nervous system that can partially bypass the BBB (16, 19). After administration to the nasal mucosa, therapeutic agents may reach the brain through several non-mutually exclusive routes, including olfactory nerve-associated transport, trigeminal nerve-associated transport, epithelial or paracellular absorption across the nasal mucosa, and subsequent movement through cerebrospinal fluid, perivascular spaces, or lymphatic-like drainage pathways (20, 21). These routes are especially relevant for glioma therapy because they may allow repeated, noninvasive administration of drugs or nanoparticles without requiring direct intracranial injection. Recent preclinical studies have demonstrated that intranasal nanocarriers can achieve brain or glioma-associated delivery and produce therapeutic effects in intracranial glioma models (17, 22). The olfactory pathway is particularly important for transport from the upper nasal cavity toward the olfactory bulb and anterior brain regions. Nanoparticles deposited in the olfactory region may be retained in the mucus layer, interact with olfactory epithelium, and move along neuronal or perineural pathways. The trigeminal pathway may provide additional access to the brainstem, meninges, and deeper brain structures. Although the relative contribution of each pathway depends on particle size, surface charge, mucoadhesion, formulation viscosity, and administration technique, these anatomical routes provide a biological basis for nose-to-brain delivery (23). Importantly, intranasal delivery does not eliminate all biological barriers; rather, it changes the barrier problem from systemic BBB penetration to nasal deposition, mucosal retention, epithelial transport, and brain distribution (24).

Several recent experimental studies support the feasibility of intranasal nanomedicine for glioma therapy. Sandbhor et al. showed that targeted nano-delivery of chemotherapy through the intranasal route suppressed *in vivo* GBM growth and prolonged survival in an intracranial mouse model, supporting the feasibility of achieving therapeutically relevant intracranial delivery through the nasal route. However, such efficacy should not by itself be interpreted as evidence that intranasal administration provides lower systemic exposure or superior pharmacokinetics than systemic delivery unless direct route-to-route comparisons are available (25). Marrocco et al. further demonstrated that ferritin-based nanovectors could mediate nose-to-brain selective drug delivery to glioma, reduce tumor growth, and improve survival in glioma-bearing mice (22). These studies are important because they provide direct preclinical evidence that intranasal nanocarriers can reach intracranial glioma tissue and produce survival benefit. More recent formulation studies have expanded the diversity of intranasal glioma nanoplatforms. Ferreira et al. developed biomimetic nanoparticles for nose-to-brain GBM-targeted therapy and showed that such platforms can promote nasal mucosal permeation, controlled drug release, and tumor-cell-associated targeting features (17). Koh et al. reported that intranasal administration of temozolomide-conjugated nanoparticles combined with transcranial magnetic stimulation improved survival in GBM xenograft models, suggesting that intranasal nanomedicine may also be integrated with physical enhancement strategies to improve brain tumor therapy (26). Although these studies mainly focus on chemotherapy rather than immunotherapy, they provide the delivery foundation required for the development of intranasal nano-immunotherapeutic systems. For immunotherapy, the importance of intranasal delivery lies in its potential to localize immune modulation within the brain tumor microenvironment. Instead of exposing the whole body to high levels of immune agonists or checkpoint-modulating nucleic acids, intranasal nanocarriers may allow repeated delivery of immune-active payloads to intracranial tumor sites. This is particularly relevant for potent innate immune agonists, siRNA, CRISPR systems, and immunostimulatory nanostructures, which require protection from degradation and controlled spatial release. Therefore, the intranasal route provides a rational platform for transforming brain delivery from a passive pharmacokinetic challenge into an active immune-engineering strategy.

Nano-immunotherapy provides the conceptual bridge between intranasal delivery and glioma immune microenvironment reprogramming. Traditional drug delivery focuses primarily on whether a therapeutic agent reaches the tumor. In contrast, nano-immunotherapy asks whether the delivered payload can reshape the immune contexture of the tumor after arrival. This distinction is crucial in GBM, where therapeutic failure is driven not only by tumor-cell survival but also by myeloid dominance, T-cell exclusion and dysfunction, checkpoint activation, poor antigen presentation, and insufficient innate immune stimulation (27, 28). Nanocarriers can be engineered to carry immune agonists, siRNA, immune checkpoint modulators, chemokine-axis inhibitors, gene-editing systems, or combination payloads that act on multiple components of the glioma ecosystem (29). Two recent studies are particularly important for framing intranasal nano-immunotherapy in glioma. Mahajan et al. developed cGAS-agonistic spherical nucleic acids that reprogrammed the GBM immune microenvironment and promoted antitumor immunity, highlighting the potential of nanostructured innate immune agonists to convert an immunologically cold, myeloid-enriched tumor into a more immune-responsive state (18). This work is important because it links nanotechnology with cGAS-STING pathway activation, myeloid reprogramming, T-cell trafficking, and antitumor immunity. It supports the idea that nanomedicine can be used not only for drug delivery but also for immunological remodeling.

Tang et al. provided an even more direct example of intranasal glioma nano-immunotherapy by constructing a self-assembled nanomicelle based on a brain tumor-targeting T7 peptide for siRNA delivery through intranasal administration. This study is highly relevant because it integrates three key elements of the present review: nose-to-brain delivery, nanocarrier-mediated nucleic acid protection, and glioma immunotherapy. The T7 peptide design supports brain tumor targeting, while the nanomicelle structure facilitates siRNA delivery and improves the feasibility of noninvasive administration. Together with cGAS-agonistic spherical nucleic acids, this work provides a foundation for discussing how intranasal nanoplatforms can deliver immune-modulating payloads to glioma tissue.

Beyond these directly relevant examples, several systemic or locally delivered nano-immunotherapeutic studies help define the immune mechanisms that future intranasal platforms may exploit. CXCR4-targeted nitric oxide nanoparticles delivering PD-L1 siRNA have been used for GBM immunotherapy, supporting the strategy of combining tumor targeting, nitric oxide biology, and immune checkpoint silencing (30). CXCR4/CXCL12-blocking synthetic protein nanoparticles have been used to enhance glioma immunotherapy by disrupting a major chemokine-mediated immunosuppressive axis (31). Nanoparticle-mediated blockade of CXCL12/CXCR4 signaling has also been combined with MRI radiomics to monitor early therapeutic responses, emphasizing the importance of imaging-based translational validation (13). These studies are not all intranasal, but they identify immune targets and mechanistic strategies that can be adapted to intranasal nanocarrier design.

Therefore, the rationale for intranasal nano-immunotherapy rests on a layered evidence base. First, intranasal nanocarriers can improve nose-to-brain delivery and produce therapeutic benefit in intracranial glioma models. Second, nanocarriers can deliver immune-active payloads capable of reprogramming the GBM immune microenvironment. Third, intranasal siRNA nanomicelles demonstrate that these two concepts can be combined into a noninvasive glioma immunotherapy strategy (32). However, current evidence remains fragmented, and this fragmentation is also reflected in the existing review literature. Previous reviews have usually discussed nose-to-brain delivery, glioma nanomedicine, or glioma immunotherapy as separate topics. Reviews focused on intranasal delivery have mainly emphasized anatomical transport routes, formulation properties, and pharmacokinetic advantages, but often pay less attention to immune microenvironment reprogramming in glioma (33). In contrast, reviews on glioma nano-immunotherapy have commonly discussed immune pathways, checkpoint modulation, myeloid-cell remodeling, or nanocarrier-mediated antitumor mechanisms, but have not systematically incorporated the unique requirements of intranasal administration, including nasal deposition, olfactory and trigeminal transport, mucosal safety, and dose translation. To avoid conflating route feasibility with immune efficacy, we stratify the available literature into three evidence levels throughout this review. Level I represents direct intranasal glioma nano-immunotherapy evidence, in which intranasal administration, nanocarrier-mediated delivery, and immune reprogramming are all experimentally demonstrated. Level II includes intranasal glioma nanodelivery studies that establish nose-to-brain or tumor-directed delivery and therapeutic efficacy but do not demonstrate immune reprogramming. Level III comprises non-intranasal glioma nano-immunotherapy studies that provide mechanistic or payload-level support for future intranasal adaptation. This evidence hierarchy is applied across the platform, mechanism, payload, table, and figure sections to distinguish established intranasal evidence from translational extrapolation.

## 2 Biological rationale for intranasal delivery in glioma

### 2.1 BBB, BTB, and the failure of systemic immunotherapy delivery

The BBB is formed by brain endothelial cells connected by tight junctions, supported by pericytes, astrocytic end-feet, basement membrane components, and perivascular immune-regulatory signals (34–38). Its physiological role is to maintain central nervous system homeostasis by restricting the entry of toxins, pathogens, and large circulating molecules. However, this same protective function creates a major pharmacological barrier for glioma therapy. Many immune therapeutics, including monoclonal antibodies, cytokines, nucleic acids, and immune agonists, are large, hydrophilic, unstable, or rapidly cleared, making it difficult for them to reach intracranial tumor sites after systemic administration. In GBM, the BTB is often more permeable than the intact BBB, but this permeability is uneven. The enhancing tumor core may show vascular leakage, whereas the invasive margin and infiltrative tumor cells may remain protected by a relatively intact BBB. This heterogeneity is clinically important because residual glioma cells at the invasive edge are a major source of recurrence. Therefore, a therapy that reaches only the disrupted tumor core may fail to eliminate the cells most responsible for relapse. Systemic dose escalation is not an ideal solution because it may increase peripheral toxicity without ensuring homogeneous intracranial delivery.

Systemically administered nanomedicines also face several biological obstacles before reaching glioma tissue. They may be opsonized in the circulation, cleared by the mononuclear phagocyte system, accumulate in the liver and spleen, or interact with peripheral immune cells before reaching the brain. Even when nanoparticles reach the tumor vasculature, they must cross the BTB, penetrate the dense extracellular matrix, avoid uptake by nonproductive cell populations, and release their payload in the correct immune or tumor-cell compartment (39–42). These barriers are especially problematic for immune-active payloads because their therapeutic effect depends on spatial precision. For example, a STING agonist, TLR ligand, siRNA, or checkpoint-modulating agent may have very different effects depending on whether it is delivered to tumor cells, dendritic cells, macrophages, microglia, endothelial cells, or peripheral immune tissues. Recent systemic nano-immunotherapy studies illustrate both the promise and the limitations of this approach. Alghamri et al. used synthetic protein nanoparticles to deliver an adjuvant CXCR4/CXCL12 signaling inhibitor for glioma immunotherapy, showing that nanocarrier design can help target a chemokine axis involved in immune suppression and tumor progression (31). Hsieh et al. developed CXCR4-targeted nitric oxide nanoparticles to deliver PD-L1 siRNA for GBM immunotherapy, demonstrating that targeted nanoparticles can combine tumor-homing, immune checkpoint silencing, and nitric oxide-mediated modulation (30). These studies highlight the need for active targeting and immune-specific payload design when systemic delivery is used.

Similarly, nanoparticle-mediated blockade of CXCL12/CXCR4 signaling has been shown to enhance GBM immunotherapy and can be monitored using MRI radiomics as an early response assessment strategy (13). This study is particularly relevant for translational development because it connects therapeutic delivery with noninvasive monitoring. However, these systemic strategies still need to overcome circulation-associated clearance, peripheral exposure, and heterogeneous BBB/BTB penetration. Intranasal delivery may reduce dependence on systemic circulation by exploiting anatomical connections between the nasal cavity and the central nervous system, but this theoretical advantage should not be equated with pharmacological superiority over systemic administration. A meaningful route comparison requires quantitative assessment of brain and tumor exposure, tumor-to-normal-brain or target-to-off-target ratios, plasma exposure, peripheral-organ distribution, and treatment-associated toxicity. Where direct head-to-head data are available, these parameters should be evaluated together with therapeutic efficacy. Thus, intranasal delivery should be regarded as a complementary brain-directed strategy whose advantage over systemic delivery must be demonstrated experimentally rather than inferred solely from increased whole-brain accumulation.

### 2.2 Nose-to-brain transport routes

Nose-to-brain delivery occurs through multiple anatomical and physiological pathways. The first and most commonly discussed route is the olfactory pathway. Drugs or nanoparticles deposited in the upper nasal cavity can interact with the olfactory epithelium and move toward the olfactory bulb through neuronal, perineural, or extracellular mechanisms. This pathway may be particularly useful for accessing anterior brain regions and may provide a relatively direct connection between the nasal mucosa and the central nervous system (43–46). However, successful use of this route depends on formulation properties, nasal deposition efficiency, mucosal retention time, and resistance to mucociliary clearance. The second route is the trigeminal pathway. Branches of the trigeminal nerve innervate both respiratory and olfactory regions of the nasal cavity and provide connections to the brainstem and meningeal compartments. This pathway may broaden the distribution of intranasally administered agents beyond the olfactory bulb and anterior brain. For glioma therapy, the trigeminal route is relevant because GBM may arise in or invade different brain regions, and effective therapy may require distribution beyond a single anatomical entry point (47–51). The olfactory and trigeminal routes should therefore be considered complementary rather than mutually exclusive. The third route involves mucosal absorption followed by fluid-associated distribution through cerebrospinal fluid, perivascular spaces, interstitial flow, or lymphatic-like drainage pathways. Nanoparticles may also undergo partial systemic absorption, which can contribute to brain exposure indirectly but may also increase peripheral distribution. Therefore, the pharmacokinetics of intranasal nanomedicine are complex. A well-designed intranasal glioma therapy should ideally maximize mucosal retention and brain entry while minimizing swallowing, pulmonary deposition, and systemic leakage.

Experimental glioma studies provide increasing support for these transport concepts. Sandbhor et al. demonstrated that intranasal targeted nanocarriers could suppress intracranial GBM growth and extend survival, supporting the feasibility of using nasal delivery to achieve therapeutic effects in brain tumors (25). Marrocco et al. showed that ferritin-based nanovectors could mediate selective nose-to-brain delivery to glioma and improve survival outcomes, suggesting that endogenous carrier-inspired nanostructures may be useful for brain tumor targeting (22). Ferreira et al. reported biomimetic nanoparticles designed for nose-to-brain GBM-targeted therapy, emphasizing the importance of particle engineering, nasal mucosal permeation, and tumor-associated recognition (17). Koh et al. further suggested that intranasal temozolomide-conjugated nanoparticles may be enhanced by transcranial magnetic stimulation, indicating that physical modulation could be combined with nasal nanomedicine to improve therapeutic performance (26). These studies collectively support the view that intranasal delivery can improve access to intracranial glioma models, but they also reveal several unresolved issues. First, most studies differ in nanoparticle composition, size, surface chemistry, payload, dosing frequency, and animal model, making direct comparison difficult. Second, many studies focus on tumor suppression and survival but provide limited mechanistic dissection of the exact transport route. Third, the anatomy of the rodent nasal cavity differs substantially from that of humans, particularly in the relative surface area of olfactory epithelium. Therefore, future intranasal nano-immunotherapy studies should not only demonstrate efficacy but also include biodistribution, regional brain accumulation, nasal mucosal safety, and translationally relevant dosing analyses.

Importantly, nose-to-brain delivery should not be equated with tumor-selective delivery. Reaching the brain, reaching the tumor, and reaching the relevant immune-cell compartment represent three distinct pharmacological steps. First, intranasally administered nanoparticles must deposit in the appropriate nasal region, resist mucociliary clearance, cross or bypass the nasal epithelium, and achieve measurable brain exposure (52–54). Second, brain exposure must translate into accumulation within glioma tissue, particularly infiltrative margins and tumor-associated niches rather than only the olfactory bulb, normal brain parenchyma, cerebrospinal fluid, or perivascular spaces (45, 55). Third, tumor exposure must lead to delivery of the payload to the intended cellular compartment, such as glioma cells, macrophages, resident microglia, dendritic cells, endothelial-associated immune niches, or T cells (56). Failure at any of these steps may result in brain signal without tumor efficacy, tumor exposure without immune pharmacodynamic activity, or local immune activation without meaningful antitumor benefit. Therefore, future intranasal nano-immunotherapy studies should separately validate brain biodistribution, tumor-selective accumulation, and immune-cell-specific payload delivery.

### 2.3 Why nanoparticles are needed for intranasal delivery

Nanoparticles are critical for intranasal glioma therapy because most immune-active payloads are not suitable for direct nasal administration in free form (57–61). Nucleic acids are vulnerable to enzymatic degradation, immune agonists may diffuse nonspecifically or trigger toxicity, proteins and peptides may have poor mucosal permeability, and small-molecule drugs may be rapidly cleared from the nasal cavity (62–64). Nanocarriers can protect these payloads from degradation, increase residence time on the nasal mucosa, improve epithelial transport, support brain exposure, and, when combined with appropriate targeting ligands or responsive release designs, potentially enhance tumor- or immune-cell-directed delivery after reaching intracranial sites. Intranasal delivery efficiency is influenced by three groups of nanocarrier properties: physicochemical parameters, including particle size, surface charge, and hydrophilicity; nasal-interaction properties, including mucoadhesion and mucus penetration; and targeting features, including ligand modification (52, 65, 66). Smaller particles may diffuse more readily through mucus and extracellular spaces, whereas mucoadhesive systems may prolong nasal residence but risk being trapped in mucus and cleared (67–69). Positively charged particles can interact with negatively charged mucins and cell membranes, but excessive cationic charge may increase toxicity (70–72). Biomimetic coatings, peptide ligands, ferritin-based systems, and spherical nucleic acid architectures may help balance these competing requirements (73). Therefore, intranasal nanocarrier design requires optimization not only for tumor targeting but also for nasal physiology.

The importance of nanocarrier design is illustrated by several recent glioma studies. cGAS-agonistic spherical nucleic acids demonstrate how nanostructured nucleic acid systems can be used to activate innate immune signaling and reprogram the GBM immune microenvironment (18). T7 peptide-mediated self-assembled nanomicelles show how brain tumor-targeting peptides can be integrated with siRNA delivery and intranasal administration for glioma immunotherapy (32). Ferritin-based nanovectors provide an example of protein-inspired carriers that support nose-to-brain selective delivery and survival benefit in glioma-bearing mice (22). Biomimetic nanoparticles further show how tumor-cell-associated or membrane-inspired designs may improve GBM targeting and translational potential (17). For intranasal nano-immunotherapy, nanoparticles have an additional immunological function. They can determine which immune pathway is activated, which cell type receives the payload, and how long the immune stimulus persists. For example, delivery of siRNA against PD-L1, CD47, or other immune suppressive targets requires efficient cytosolic release, whereas delivery of cGAS-STING or TLR agonists requires controlled activation of innate immune compartments. Similarly, nanocarriers that modulate CXCR4/CXCL12 signaling, nitric oxide biology, or myeloid-cell behavior must be designed to act within the tumor immune microenvironment rather than only within tumor cells. This explains why future intranasal formulations should be evaluated not only by drug concentration in the brain, but also by immune-cell infiltration, macrophage/microglia phenotypes, dendritic-cell activation, checkpoint expression, cytokine profiles, and T-cell function.

In summary, nanoparticles are needed because intranasal delivery alone cannot solve all barriers in glioma therapy. The nasal route provides access, but nanocarriers provide protection, targeting, controlled release, and immune-programming capacity. The most promising future systems will likely combine optimized nose-to-brain transport with immune-specific payloads such as STING agonists, TLR agonists, siRNA, CRISPR systems, checkpoint modulators, or chemokine-axis inhibitors (33). This combination is the central rationale for developing intranasal nano-immunotherapy as a distinct therapeutic strategy for glioma. Recent preclinical studies have demonstrated that intranasal and nose-to-brain nanoplatforms can improve intracranial drug delivery, enhance glioma targeting, and prolong survival in orthotopic or xenograft glioma models. Representative intranasal and brain-directed nanocarriers for glioma therapy are summarized in **Supplementary Table S1**. **Figure 1** illustrates the shift from blood-to-brain systemic delivery to nose-to-brain nanomedicine for glioma therapy. By integrating intranasal transport, nanocarrier protection, tumor-targeting design, and immune payload delivery, this strategy may provide a framework for testing whether brain exposure can be converted into tumor-localized and immune-cell-relevant pharmacological activity. However, each step requires separate validation, because improved brain exposure alone does not prove tumor-selective delivery or productive immune microenvironment reprogramming.

**Figure 1:**
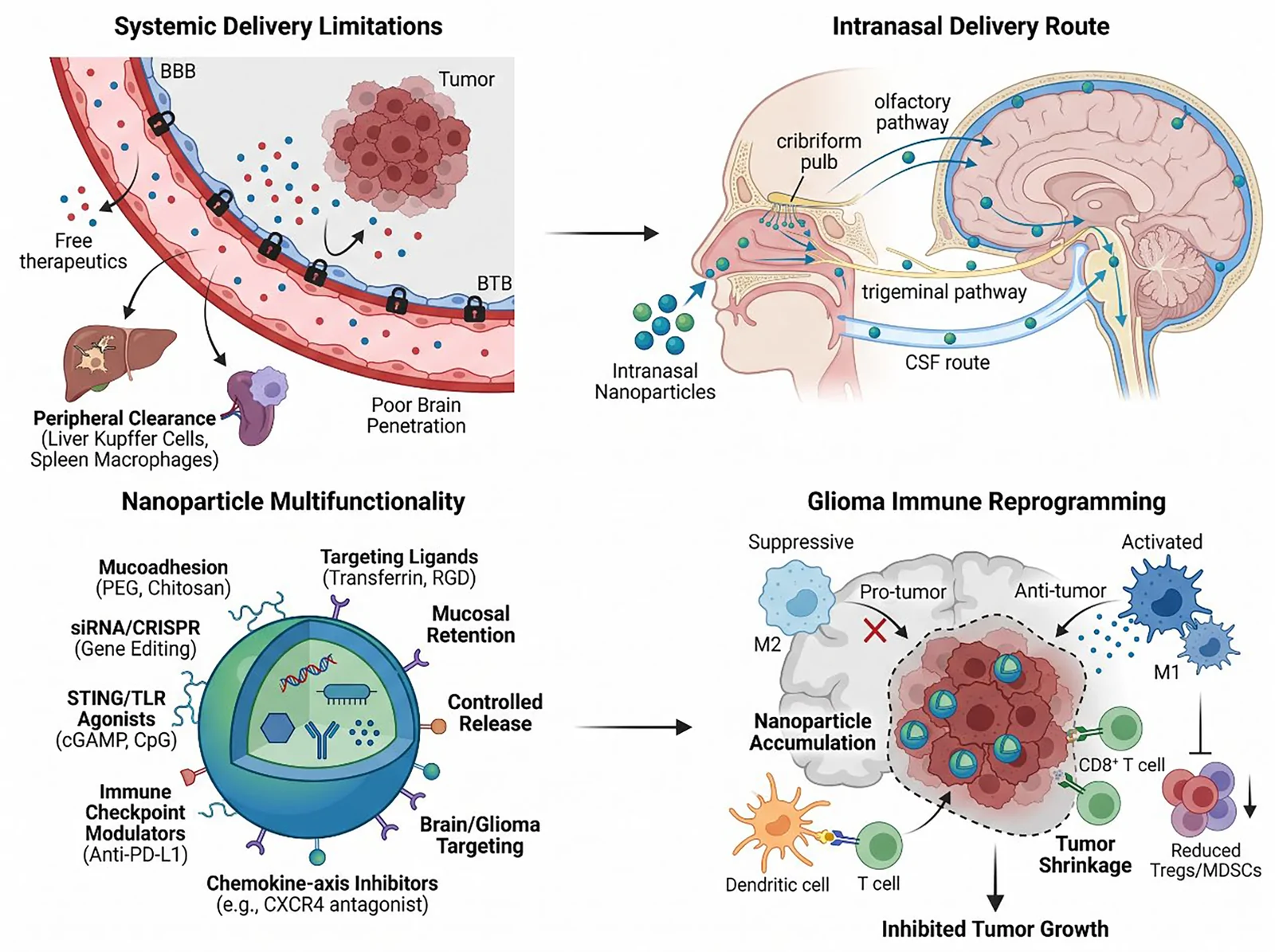
Rationale for intranasal nano-immunotherapy in glioma. Systemic nanomedicines are limited by BBB/BTB heterogeneity and peripheral clearance, whereas intranasal nanoparticles reach the brain through olfactory, trigeminal, and CSF/perivascular routes. Nanocarriers protect immune-active payloads, enhance glioma targeting, and enable controlled release to activate innate immunity, reprogram myeloid cells, increase T-cell infiltration, and promote tumor control.

### 2.4 Comparison with alternative glioma immunotherapy and brain-delivery strategies

Intranasal nano-immunotherapy should be considered a complementary strategy rather than a universal replacement for existing glioma immunotherapy or brain-delivery approaches. Each strategy addresses a different barrier in GBM therapy (74). Systemic immunotherapy has the advantage of clinical accessibility, repeatable dosing, and broad immune engagement, but its efficacy in unselected GBM remains limited by poor intracranial drug exposure, low T-cell infiltration, myeloid-dominant immunosuppression, corticosteroid use, and tumor heterogeneity (75, 76). For example, the phase III CheckMate 143 trial evaluated nivolumab versus bevacizumab in recurrent GBM and demonstrated the difficulty of translating systemic checkpoint blockade into broad clinical benefit in this disease (77). Convection-enhanced delivery directly infuses therapeutic agents into brain tissue through a catheter-generated pressure gradient (78, 79). This approach can bypass the BBB and achieve high local drug concentrations over a defined volume of distribution (80). However, it is invasive and depends on catheter placement, infusion parameters, backflow control, interstitial pressure, tumor heterogeneity, and real-time monitoring of drug distribution (81, 82). Previous reviews have emphasized that CED can expand intratumoral distribution beyond passive diffusion, but clinical reproducibility and delivery reliability remain major challenges.

Focused ultrasound-mediated BBB opening provides another emerging strategy for enhancing brain drug delivery. Compared with intranasal delivery, FUS can be spatially targeted and may transiently increase vascular permeability in selected brain regions (83, 84). Early clinical studies and systematic reviews suggest that repeated ultrasound-mediated BBB opening is feasible in GBM, including trials using implantable or MR-guided systems (85, 86). However, this strategy requires specialized equipment, microbubbles or implantable devices in some protocols, precise imaging guidance, and careful safety monitoring for edema, hemorrhage, inflammation, or off-target BBB disruption.

Local or intratumoral delivery, including drug-loaded wafers, intratumoral injection, local immunotherapy, or postsurgical depot systems, can increase drug exposure near the resection cavity or injected tumor region while reducing systemic exposure (87–89). This approach is particularly useful when the tumor is surgically accessible (90). However, local delivery is limited by invasive administration, heterogeneous tissue diffusion, short penetration distance, procedural logistics, device-related complications, and difficulty treating diffuse infiltrative margins (91, 92). Recent analyses of Gliadel wafers and intratumoral immunotherapy emphasize both the value and the practical limitations of local delivery strategies (93).

Compared with these approaches, intranasal nano-immunotherapy may offer several potential advantages: noninvasive and repeatable administration, reduced dependence on systemic circulation, compatibility with nucleic acids or immune agonists that require carrier protection, and possible access to olfactory, trigeminal, CSF-associated, and perivascular transport routes (16, 20). Nevertheless, these advantages remain largely hypothetical for glioma immunotherapy and must be validated experimentally. Intranasal delivery faces its own barriers, including variable nasal deposition, mucociliary clearance, enzymatic degradation, partial swallowing or systemic leakage, species differences in nasal anatomy, limited human dosing data, and uncertain regional brain distribution (44, 94). Therefore, the specific value of intranasal nano-immunotherapy is not that it is intrinsically superior to systemic, catheter-based, ultrasound-mediated, or local delivery. Rather, its potential niche may lie in repeated, noninvasive delivery of immune-active nanocarriers when invasive delivery is undesirable, when systemic exposure should be minimized, or when spatial immune pharmacodynamic validation supports brain-tumor-associated immune modulation (95).

Overall, future development should compare intranasal nano-immunotherapy directly with systemic, CED-based, FUS-assisted, and local delivery strategies using matched payloads, orthotopic models, quantitative biodistribution, immune pharmacodynamics, survival, recurrence, and safety endpoints. Such head-to-head comparisons are needed to determine whether intranasal delivery provides a true therapeutic advantage or only a different delivery route.

## 3 Nano-platforms for intranasal and brain-targeted glioma immunotherapy

Nanotechnology provides a flexible platform for integrating brain delivery, tumor targeting, payload protection, controlled release, and immune modulation (8, 96). In glioma, this integration is particularly important because therapeutic agents must overcome both anatomical delivery barriers and profound immune suppression within the tumor microenvironment. Intranasal administration offers a noninvasive route for nose-to-brain transport, but the therapeutic performance of this route depends heavily on the properties of the nanocarrier (16). Particle size, surface charge, ligand modification, mucoadhesion, biodegradability, and payload-release kinetics all influence whether a formulation can remain in the nasal cavity, reach the brain, accumulate in glioma tissue, and activate the intended immune mechanism (97). Importantly, these platforms differ substantially in their route-specific evidence, as illustrated by recent studies demonstrating direct intranasal immune reprogramming or intranasal glioma-targeted nanodelivery without established immune remodeling (17, 18). Accordingly, the studies discussed below are classified as Level I, direct intranasal glioma nano-immunotherapy; Level II, intranasal glioma nanodelivery without demonstrated immune reprogramming; or Level III, non-intranasal glioma nano-immunotherapy supporting future intranasal adaptation. This distinction is maintained throughout this section to prevent mechanistic evidence obtained through systemic or local administration from being interpreted as established intranasal efficacy. To minimize redundancy, this section focuses primarily on carrier architecture, administration route, payload compatibility, and delivery-related advantages and limitations. Immune mechanisms are synthesized across platforms in Section 4, whereas comparative translational readiness is addressed in Section 5.

### 3.1 Spherical nucleic acids and immunostimulatory nucleic acid nanostructures

Spherical nucleic acids and related immunostimulatory nucleic acid nanostructures are attractive for glioma immunotherapy because they combine nanoscale delivery with innate immune activation (98–100). Unlike free nucleic acids, which are vulnerable to enzymatic degradation and poor cellular uptake, nanostructured nucleic acids can improve stability, promote intracellular delivery, and enhance interactions with innate immune sensing pathways. This is especially relevant for GBM, where defective antigen presentation, poor dendritic-cell activation, and dominant myeloid suppression contribute to a “cold” immune phenotype. From a delivery perspective, immunostimulatory nucleic-acid nanoplatforms include cGAS-agonistic spherical nucleic acids, sequential-release STING-restoring systems, and poly(I)-loaded lipid nanoparticles. These platforms share an ability to protect nucleic-acid or immune-stimulatory cargos and facilitate intracellular immune-sensor engagement, but they differ substantially in carrier architecture, release kinetics, and intracellular requirements. Importantly, the representative systems reported to date were not developed as intranasal glioma formulations and therefore primarily provide Level III evidence for future route adaptation. Their principal translational challenges include control of regional brain exposure, dose-dependent inflammatory toxicity, reproducible intracellular delivery, and the need to distinguish productive antitumor immune activation from nonspecific neuroinflammation. The corresponding immune mechanisms are synthesized in Section 4.1 rather than repeated here.

STING signaling can also be therapeutically restored or amplified through more complex release systems. Yu et al. reported a sequential-release HydroLipo system for STING gene epigenetic reprogramming and immune activation in GBM (101). This platform combined hydrogel and nanoparticle components to achieve controlled release, with the goal of correcting STING signaling dysfunction and enhancing immune activation. Although this system is not primarily an intranasal platform, it supports a broader principle: glioma immune resistance may arise not only from insufficient delivery of immune agonists, but also from tumor-intrinsic defects in immune sensing (102–106). Therefore, future intranasal systems may need to combine brain delivery with epigenetic priming, innate immune agonism, and controlled local release. Another important nucleic acid strategy is delivery of double-stranded RNA mimetics such as polyinosinic/polycytidylic acid, which can activate TLR3 and related antiviral immune programs. Brüßeler et al. showed that polyinosinic/polycytidylic lipid nanoparticles enhanced immune-cell infiltration and improved survival in a GBM mouse model (107). This study supports the idea that nanocarrier-assisted delivery of nucleic acid immune agonists can transform the immune landscape of glioma. For intranasal therapy, lipid nanoparticles carrying TLR3 agonists or related nucleic acids could be adapted to exploit nose-to-brain transport while stimulating local innate immunity within glioma-associated immune niches.

From a platform perspective, immunostimulatory nucleic acid nanostructures have high immune potency and strong payload-protection capacity, but their translational readiness is limited by inflammatory safety concerns, uncertain regional brain distribution, and the need for controlled release after intranasal administration. Compared with peptide-guided micelles or protein-based carriers, these systems may provide stronger innate immune activation but require stricter dose control and neuroinflammatory safety evaluation.

### 3.2 Peptide-guided nanomicelles and siRNA delivery

Beyond the directly relevant intranasal T7 peptide-guided nanomicelle (32), several non-intranasal nucleic-acid platforms illustrate the broader payload compatibility of glioma nanomedicine (14). These include CXCR4-targeted nanoparticles carrying PD-L1 siRNA (30), tumor-microenvironment-responsive CRISPR-Cas9 nanoparticles targeting GDF15 (108), and engineered bacterial outer-membrane vesicles co-delivering doxorubicin and CD47-siRNA (109). Rather than representing equivalent delivery strategies, these systems impose different intracellular requirements: siRNA requires efficient cytosolic release and transient target suppression, CRISPR requires controlled genome-editing activity with stringent off-target assessment, and CD47-siRNA co-delivery couples tumor-cell target modulation with downstream phagocytic responses. Because these non-intranasal platforms have not established intranasal delivery, they are considered Level III evidence for future adaptation. Their immune consequences are compared in Section 4.

### 3.3 Biomimetic and cell membrane-coated nanoparticles

Biomimetic and cell-membrane camouflaged nanoparticles are engineered to mimic native biological architectures for enhanced circulatory stability, immune compatibility, tumor targeting, and tissue penetration. In glioma therapy, biomimetic design may involve coating nanoparticles with tumor-cell membranes, immune-cell membranes, bacterial vesicles, extracellular vesicles, or other bioinspired interfaces (110–113). These coatings can provide adhesion molecules, antigenic features, immune recognition patterns, or homotypic targeting properties that are difficult to achieve with purely synthetic materials. Ferreira et al. reported biomimetic nanoparticles for nose-to-brain GBM-targeted therapy (17). Although the study mainly supports the delivery side of the present review, it is important because it shows that biomimetic design can be integrated with intranasal administration. In the context of glioma, such systems may help improve nasal mucosal interaction, brain entry, and tumor-associated targeting. Future biomimetic intranasal systems could be further loaded with immune agonists, siRNA, checkpoint modulators, or metabolic regulators to achieve both brain delivery and immune reprogramming.

Wang et al. developed biomimetic nanoparticles that directly remodeled the immunosuppressive GBM microenvironment and boosted glioma immunotherapy (73). This work supports a central concept of nano-immunotherapy: the therapeutic goal is not only to deliver drugs to tumor cells but also to alter the immune ecology of the tumor. Biomimetic nanoparticles can be engineered to modulate antigen presentation, macrophage polarization, T-cell infiltration, and immune checkpoint responsiveness. Recent advances in biomimetic and personalized nanovaccines further extend this concept by integrating tumor-associated or patient-specific antigens with biologically inspired nanocarriers to enhance antigen presentation and adaptive immune priming (114). Such platforms may incorporate extracellular vesicles, immune-cell membrane coatings, artificial antigen-presenting systems, mRNA or neoantigen cargos, and innate immune adjuvants such as TLR or STING agonists. Although these approaches have not yet established intranasal efficacy in glioma, they provide Level III mechanistic support for future intranasal nanovaccine strategies aimed at coupling brain-directed delivery with antigen-specific antitumor immunity.

Such platforms are particularly attractive for GBM because the tumor microenvironment is dominated by macrophages and microglia, which can either suppress or support antitumor immunity depending on their state. Extracellular vesicles represent another biomimetic strategy (115–119). Tian et al. used engineered extracellular vesicles to enhance immune checkpoint inhibition in radiation-primed GBM (120). This strategy is important because radiation can increase antigen release, modulate checkpoint expression, and alter immune-cell recruitment, while engineered vesicles can deliver immune-modulating signals within this primed microenvironment. The combination of radiation and EV-based immunotherapy suggests that biomimetic platforms may be most effective when paired with treatments that expose tumor antigens or reshape the local immune niche.

Engineered bacterial outer membrane vesicles are especially interesting because they combine delivery capacity with intrinsic immunostimulatory activity. You et al. showed that OMV-based co-delivery of doxorubicin and CD47-siRNA could overcome immune resistance in GBM immunotherapy (109). OMVs can act as carriers, immune adjuvants, and pattern-recognition stimuli. However, their immunogenicity is a double-edged sword. While it may enhance antitumor immunity, it may also increase the risk of excessive inflammation, neurotoxicity, or systemic immune activation. Overall, stimuli-responsive and multimodal systems are less mature for intranasal translation than simpler micelles, lipid nanoparticles, or protein-based carriers, because they often require external activation, complex manufacturing, or lesion-specific conditions. Their value lies in mechanistic synergy rather than route feasibility alone. Therefore, their future adaptation to intranasal delivery should be justified by whether the added modality improves immune pharmacodynamics, tumor selectivity, or safety compared with simpler nanocarriers.

### 3.4 Ferritin, protein nanoparticles, and endogenous carrier-inspired systems

Protein-based nanoparticles and endogenous carrier-inspired systems offer a biologically compatible strategy for glioma delivery. Ferritin, albumin, transferrin-related systems, and synthetic protein nanoparticles can be engineered to carry drugs, nucleic acids, or immune modulators while preserving favorable biocompatibility (121–124). Compared with some synthetic polymers or inorganic materials, protein nanocarriers may have lower long-term toxicity and better biodegradability. They can also be modified with targeting ligands or designed to exploit receptor-mediated transport pathways. Ferritin-based nanovectors are particularly relevant to intranasal glioma therapy (125–129). Marrocco et al. demonstrated that ferritin nanovectors enabled nose-to-brain selective drug delivery to glioma, reduced tumor growth, and improved survival in preclinical models (22). This study supports the use of endogenous carrier-inspired nanoplatforms for noninvasive brain tumor delivery. Although the immune mechanism was not the primary focus, ferritin-based intranasal systems could be adapted to carry immune-active payloads such as STING agonists, TLR agonists, siRNA, or macrophage-modulating agents.

Synthetic protein nanoparticles have also been used for glioma immunotherapy. Alghamri et al. developed a systemically delivered adjuvant CXCR4/CXCL12 signaling inhibitor encapsulated in synthetic protein nanoparticles for glioma immunotherapy (31). The CXCL12/CXCR4 axis is important because it contributes to tumor invasion, immune-cell trafficking, and immunosuppressive niche formation. Blocking this axis may reduce immune exclusion and enhance responsiveness to immunotherapy. This study illustrates how protein-like nanoparticles can be used to target chemokine-driven immune suppression in glioma. In a related strategy, nanoparticle-mediated blockade of CXCL12/CXCR4 signaling was shown to enhance GBM immunotherapy, with MRI radiomics used to monitor early treatment response (13). This study is valuable because it links nanotherapeutic design with noninvasive response assessment. For clinical translation, early imaging biomarkers are essential because immune-related responses in GBM may be difficult to distinguish from edema, pseudoprogression, or radiation-induced changes. Integrating nanotherapy with MRI radiomics may help bridge preclinical efficacy and clinical monitoring. Endogenous carrier-inspired nanoparticles therefore occupy an important position between delivery engineering and translational feasibility. Their biological origin or protein-like architecture may improve compatibility, while their modular design allows immune payload loading and surface modification. For intranasal nano-immunotherapy, ferritin and protein nanoparticles may be especially useful because they can combine mucosal delivery, brain targeting, and immune payload delivery. Future studies should evaluate whether these carriers can maintain stability in nasal secretions, avoid rapid mucociliary clearance, reach orthotopic glioma tissue, and induce measurable immune remodeling.

### 3.5 Stimuli-responsive and multimodal nano-immunotherapy systems

Stimuli-responsive and multimodal nano-immunotherapy systems are designed to respond to internal or external cues, including radiation, light, redox status, pH, enzymes, metal ions, oxidative stress, or magnetic fields. These systems are attractive for glioma because the tumor microenvironment is heterogeneous and difficult to treat with a single modality. By integrating chemotherapy, radiotherapy, photodynamic therapy, immunotherapy, and metabolic modulation, multimodal nanoplatforms may induce tumor-cell death while simultaneously activating antitumor immunity. Zhu et al. developed a bacteria-mediated metformin-loaded peptide hydrogel that reprogrammed the GBM immune microenvironment (130). This study illustrates how metabolic modulation, bacterial components, and biomaterial-based delivery can be combined to alter tumor immunity. Although this is not an intranasal platform, it supports the broader principle that glioma immunotherapy may benefit from local or semi-local delivery systems that reshape the metabolic and immune microenvironment. Metformin-associated metabolic stress may influence immune suppression, while bacterial elements may provide immune stimulation.

Radiation-responsive nanoplatforms provide another important strategy. Wang et al. developed an engineered nanoplatform with tropism toward irradiated GBM that augmented radioimmunotherapy efficacy (131). Radiotherapy can induce immunogenic cell death, release tumor antigens, increase inflammatory signaling, and alter vascular or stromal properties. A nanoplatform that selectively accumulates in irradiated tumor tissue can exploit these changes to improve specificity and reduce immune-related adverse events. This strategy is highly relevant to GBM because radiotherapy remains a standard component of treatment and could be used to prime the tumor for nano-immunotherapy. Cuproptosis-based nano-immunotherapy is a newer direction. Chen et al. developed a copper-coordination driven brain-targeting nanoassembly for GBM immunotherapy by cuproptosis-mediated tumor immune microenvironment reprogramming (132). Cuproptosis may induce tumor-cell stress and death in a manner that promotes immune activation. Copper-based systems may also affect oxidative metabolism, mitochondrial function, and inflammatory signaling. In glioma, where metabolic plasticity and immune suppression are closely linked, cuproptosis-based nanomedicine offers a potentially powerful route to connect metabolic vulnerability with immune reprogramming.

Oxidative and nitrosative stress regulation can also support immunotherapy. Lee et al. reported nitric oxide/reactive oxygen species-scavenging nanoparticles that shielded T cells and potentiated anti-PD-1 therapy in GBM (133). This strategy is conceptually distinct from platforms that simply increase inflammation. Instead, it protects effector immune cells from hostile oxidative conditions within the tumor microenvironment. This is important because T-cell dysfunction in GBM is driven not only by checkpoint signaling but also by metabolic stress, hypoxia, reactive oxygen species, and suppressive myeloid cells (134–139). Photodynamic immunotherapy provides another multimodal strategy. Yan et al. developed a β-ketoenamine covalent organic framework nanoplatform combined with immune checkpoint blockade through photodynamic immunotherapy to inhibit GBM progression (140). Photodynamic therapy can induce tumor-cell damage, antigen release, and immune activation, while checkpoint blockade can sustain T-cell-mediated killing. COF-based nanoplatforms are attractive because of their porous structure, high loading capacity, and tunable photophysical properties. For brain tumors, however, light penetration and delivery route remain important translational challenges.

Overall, stimuli-responsive and multimodal systems are likely to become increasingly important for glioma nano-immunotherapy. Their strength lies in their ability to generate local tumor damage, release antigens, activate innate immunity, protect effector immune cells, and combine with checkpoint blockade. For intranasal development, the next step is to adapt these multimodal payloads into formulations that can be safely delivered through the nasal route and reach intracranial tumor sites. Such systems should be tested in clinically relevant orthotopic models and evaluated for biodistribution, immune activation, survival benefit, recurrence control, and neurotoxicity. **Figure 2** summarizes the major nanoplatforms that can support intranasal delivery to glioma and illustrates the sequential transition from nose-to-brain transport to tumor-associated payload release. These platforms provide a delivery basis for subsequent immune modulation, although the strength of route-specific immune evidence differs among systems.

**Figure 2:**
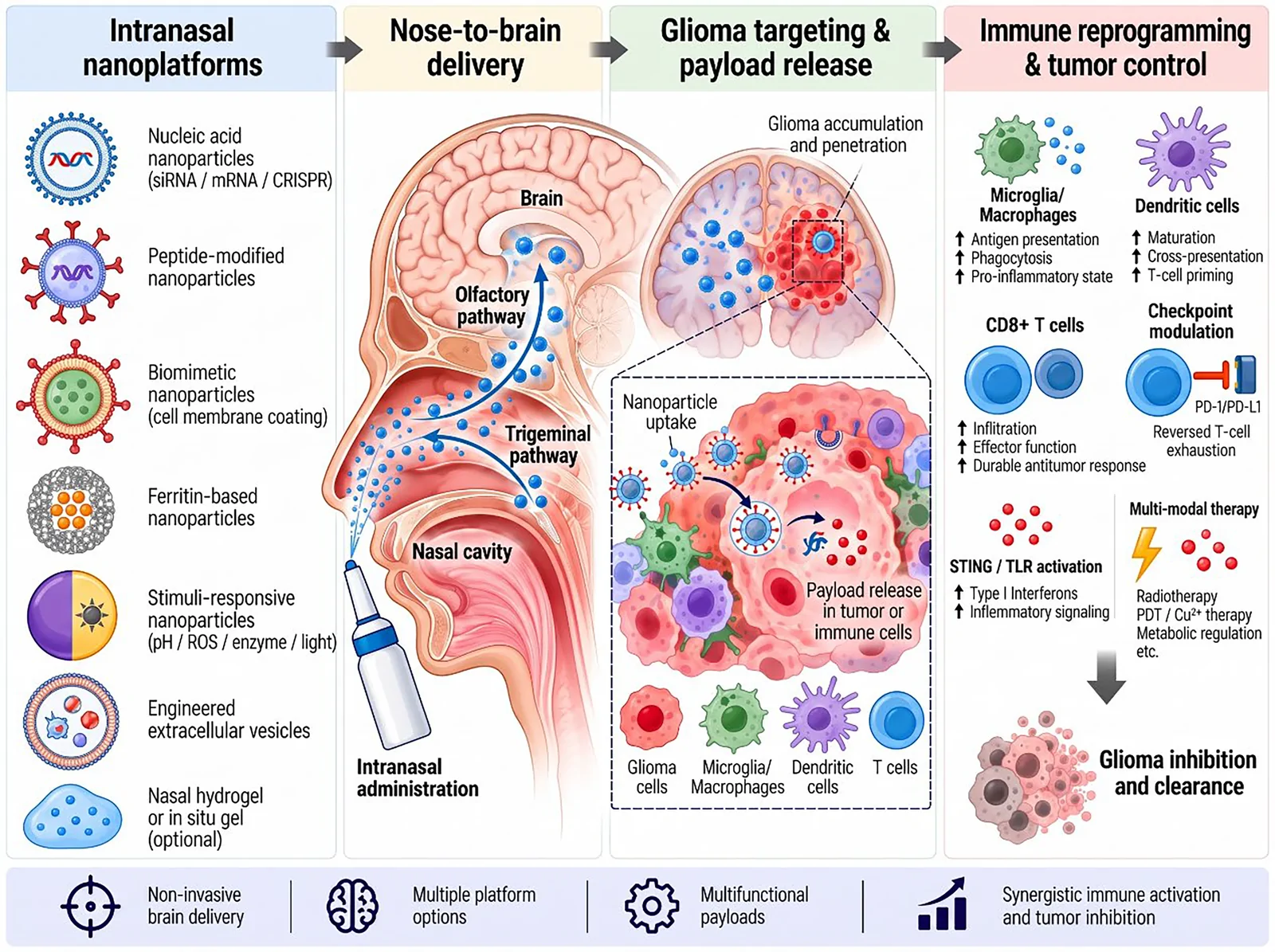
Intranasal nanoplatforms for glioma delivery and immune modulation. Representative nanocarriers include nucleic-acid nanoparticles, peptide-modified systems, biomimetic nanoparticles, ferritin-based carriers, stimuli-responsive nanoparticles, and engineered vesicles. Following intranasal administration, these platforms may access the brain through olfactory and trigeminal pathways, accumulate in glioma tissue, release therapeutic payloads, and modulate tumor-associated immune cells, ultimately supporting antitumor activity.

## 4 Immunological mechanisms: from delivery to immune microenvironment reprogramming

Because several platforms converge on overlapping immune pathways, this section synthesizes mechanisms across nanocarrier classes rather than reintroducing each study in detail. The same platform may contribute to more than one immune process; for example, an OMV-based co-delivery system may induce tumor-cell stress, promote phagocytosis, and provide innate immune stimulation, whereas a CXCL12/CXCR4-targeted system may influence both myeloid positioning and T-cell entry (31, 109). Therefore, the following subsections organize the literature by immune function rather than by individual nanoplatform. The therapeutic value of intranasal nano-immunotherapy depends not only on whether nanoparticles reach glioma tissue, but also on whether they induce productive immune remodeling after arrival (9). GBM is characterized by multiple layers of immune resistance, including weak innate immune activation, abundant suppressive myeloid cells, poor T-cell infiltration, checkpoint-mediated exhaustion, metabolic stress, and limited immunogenic tumor-cell death (141). Therefore, the goal of nano-immunotherapy is to convert delivery into immune reprogramming. This process may involve activation of innate immune sensing, modulation of astrocyte–microglia–perivascular immune-cell crosstalk, reversal of myeloid suppression, enhancement of T-cell function, and induction of immunogenic cell death. Because the mechanistic literature summarized in this section includes all three evidence levels, demonstration of an immune mechanism after systemic or local nanotherapy should not be interpreted as evidence that the same mechanism has been achieved after intranasal administration. Level I evidence directly links intranasal delivery to immune modulation, whereas Level II and Level III studies respectively support route feasibility and mechanistic plausibility.

### 4.1 Activation of innate immune sensing: cGAS-STING, TLR3, and interferon signaling

Innate immune sensing is a key entry point for converting immune-cold glioma into a more inflamed and therapy-responsive tumor. Pathways such as cGAS-STING and TLR3 can detect cytosolic DNA or double-stranded RNA-like signals, leading to type I interferon production, inflammatory chemokine release, dendritic-cell activation, and enhanced antigen presentation (142–144). In GBM, where spontaneous T-cell priming is often weak, activation of innate immune sensing may be necessary to initiate a broader antitumor immune cascade. cGAS-agonistic spherical nucleic acids provide one of the clearest examples of this approach. Mahajan et al. showed that cGAS-agonistic SNAs could reprogram the GBM immune microenvironment and promote antitumor immunity (18). Mechanistically, this type of platform is important because it uses nanostructured nucleic acid architecture to deliver or present innate immune agonism more efficiently than free agonists. By stimulating cGAS-related signaling, the platform can promote interferon-associated immune activation, enhance inflammatory communication between tumor and immune cells, and support antitumor immune responses.

STING pathway dysfunction may also need to be corrected before immune activation becomes effective. Yu et al. developed a sequential-release HydroLipo system designed for STING gene epigenetic reprogramming and immune activation in GBM (101). This approach suggests that glioma immune resistance can involve both insufficient immune stimulation and epigenetic silencing of immune-sensing machinery. Therefore, effective nano-immunotherapy may require a sequential design: first restoring immune pathway competence, and then delivering immune agonists to activate the restored pathway. TLR3 activation by polyinosinic/polycytidylic acid represents another innate immune strategy. Brüßeler et al. showed that polyinosinic/polycytidylic lipid nanoparticles enhanced immune-cell infiltration and improved survival in a GBM mouse model (107). This supports the use of LNPs to deliver RNA-like immune agonists capable of inducing antiviral-like inflammatory programs within the tumor. Such approaches may increase dendritic-cell activation, chemokine expression, and T-cell recruitment. For intranasal application, TLR3 agonist-loaded LNPs may be attractive because they can be engineered for mucosal stability and brain delivery.

However, innate immune activation must be carefully balanced. Too little activation may fail to overcome glioma immune suppression, while excessive activation may cause neuroinflammation, edema, neuronal damage, or systemic cytokine toxicity. This is particularly important for intranasal administration, where the nasal mucosa, olfactory region, and brain may all be exposed to immune-active materials. Future studies should therefore evaluate local interferon responses, inflammatory cytokines, microglial activation, brain edema, olfactory toxicity, and behavioral safety in addition to antitumor efficacy.

### 4.2 Astrocyte–resident microglia–perivascular immune-cell crosstalk after intranasal nanoparticle delivery

Intranasal nano-immunotherapy should not be interpreted only as a strategy that delivers immune payloads to tumor cells or infiltrating lymphocytes. Because nose-to-brain delivery may involve olfactory, trigeminal, cerebrospinal fluid, and perivascular transport routes, intranasally administered nanoparticles may first interact with CNS interface compartments, including the olfactory bulb, perivascular spaces, meningeal immune regions, and glioma invasive margins (53, 145). These anatomical routes create opportunities for nanoparticles to modulate not only M2-like macrophages, regulatory T cells, or exhausted CD8+ T cells, but also astrocytes, resident microglia, and perivascular immune cells. This distinction is important because glioma immunity is shaped by spatially organized cellular niches rather than by isolated immune-cell populations.

Astrocytes are active regulators of the glioma immune microenvironment. Reactive astrocytes can influence glioma invasion, vascular remodeling, extracellular-matrix organization, cytokine production, and immune-cell recruitment (146, 147). After intranasal nanoparticle delivery, immune-active payloads such as STING agonists, TLR agonists, siRNA, or chemokine-axis inhibitors may indirectly affect astrocyte function by changing local interferon signaling, inflammatory chemokines, oxidative stress, or tumor-cell damage. Conversely, astrocyte-derived mediators may regulate microglial activation, endothelial permeability, perivascular immune-cell retention, and T-cell access to glioma tissue (148, 149). Therefore, astrocytes should be considered an important intermediate cell type linking nanoparticle delivery to immune remodeling in the brain tumor niche.

Resident microglia also require specific attention because they are not identical to bone marrow-derived macrophages. Microglia are CNS-resident immune cells positioned to sense local injury, tumor-derived signals, and nanoparticle-induced innate immune activation. Intranasally delivered nanoparticles that activate cGAS-STING or TLR pathways may promote microglial inflammatory activation, antigen-presentation programs, and phagocytic activity, whereas uncontrolled stimulation may induce neuroinflammation or tissue injury. In glioma, resident microglia and infiltrating macrophages may occupy different spatial niches and perform distinct functions (75, 150, 151). Therefore, future studies should distinguish microglia from monocyte-derived macrophages rather than relying only on broad TAM or M2-like macrophage markers.

Perivascular immune cells represent another key compartment for intranasal nano-immunotherapy. The perivascular space is closely connected to brain fluid transport, vascular permeability, immune-cell trafficking, and glioma invasion (152, 153). Nanoparticles entering the brain through CSF-associated or perivascular routes may accumulate near endothelial cells, pericytes, astrocytic end-feet, perivascular macrophages, and recruited myeloid cells. Modulation of this niche may influence CXCL12/CXCR4 signaling, myeloid-cell positioning, T-cell entry, antigen presentation, edema, and treatment-related inflammation (31, 154, 155). Thus, perivascular immune remodeling may be a critical bridge between nose-to-brain delivery and functional antitumor immunity.

These interactions suggest that intranasal nano-immunotherapy should be evaluated as a spatial immune-regulatory strategy. In addition to measuring M2-like macrophages, regulatory T cells, and exhausted CD8+ T cells, future studies should assess astrocyte activation, resident microglial states, perivascular macrophage accumulation, endothelial–immune interactions, and spatial proximity among glioma cells, glial cells, and immune cells. Useful markers may include GFAP, AQP4, C3, and S100β for astrocyte responses; TMEM119, P2RY12, Sall1, Iba1, CD68, MHC-II, CD86, CD206, and CD163 for microglia/macrophage states; and CD31, PDGFRβ, LYVE1, CD206, CXCR4, CXCL12, and MHC-II for perivascular immune niches. Spatial transcriptomics, multiplex immunofluorescence, single-cell RNA sequencing, and flow cytometry should be combined to determine whether intranasally delivered nanoparticles produce beneficial immune reprogramming or nonspecific neuroinflammation.

### 4.3 Reversal of myeloid-mediated immunosuppression

Myeloid cells are central to the immunosuppressive GBM microenvironment. Tumor-associated macrophages, resident microglia, bone marrow-derived macrophages, and myeloid-derived suppressor cells can promote tumor growth, angiogenesis, invasion, matrix remodeling, and T-cell dysfunction. In many GBM tumors, myeloid cells represent a dominant immune compartment, while cytotoxic T cells are relatively scarce or dysfunctional (156–160). Importantly, these myeloid populations should be interpreted within the spatial context of the CNS tumor niche, where resident microglia, perivascular macrophages, astrocytes, endothelial cells, and infiltrating lymphocytes interact dynamically rather than functioning as isolated suppressive populations. Therefore, successful nano-immunotherapy must either reprogram suppressive myeloid cells or reduce their ability to exclude and inhibit antitumor lymphocytes. Biomimetic nanoparticles provide one strategy for direct remodeling of the myeloid-rich tumor microenvironment. Wang et al. showed that biomimetic nanoparticles could directly remodel the immunosuppressive GBM microenvironment and improve immunotherapy efficacy (73). This study supports the concept that nanoparticles can be designed to act on immune architecture rather than only on tumor cells. Rather than inducing a binary M1-to-M2 or M2-to-M1 switch, effective nano-immunotherapy should be evaluated according to lineage-resolved and functionally defined myeloid-state changes. Resident microglia, monocyte-derived macrophages, perivascular macrophages, and other infiltrating myeloid populations can occupy distinct transcriptional, metabolic, phagocytic, antigen-presenting, and immunosuppressive states within GBM. Therefore, therapeutic efficacy should be defined by changes in these state-specific programs and their spatial organization rather than by shifts in a limited set of conventional polarization markers.

The CXCL12/CXCR4 axis is another important myeloid and stromal immune-regulatory pathway. Alghamri et al. used synthetic protein nanoparticles to deliver an adjuvant CXCR4/CXCL12 signaling inhibitor for glioma immunotherapy (31). CXCL12/CXCR4 signaling can contribute to immune exclusion, tumor-cell invasion, and recruitment or retention of suppressive cells. Blocking this axis may improve immune-cell access to the tumor and reduce suppressive myeloid programs (161–165). Similarly, nanoparticle-mediated blockade of CXCL12/CXCR4 signaling enhanced GBM immunotherapy and was monitored using MRI radiomics (13). These studies support the idea that chemokine-axis modulation can connect immune reprogramming with translational imaging. Metabolic and bacterial platforms may also reshape myeloid immunity. Zhu et al. reported that a bacteria-mediated metformin-loaded peptide hydrogel reprogrammed the GBM immune microenvironment (130). This type of strategy may influence myeloid-cell metabolism, inflammatory signaling, and tumor-cell stress. Bacterial components can provide innate immune stimulation, while metformin may alter metabolic conditions that support immunosuppression. Although this platform differs from intranasal nanoparticles, it shows that immune reprogramming can be achieved by combining metabolic and microbial cues.

Cuproptosis-mediated nano-immunotherapy may further contribute to myeloid remodeling. Chen et al. developed a copper-coordination driven brain-targeting nanoassembly that induced GBM immunotherapy through cuproptosis-mediated tumor immune microenvironment reprogramming (132). Tumor-cell death induced by metabolic or mitochondrial stress may release danger signals, enhance antigen availability, and alter macrophage or microglial behavior. This connects regulated cell death with immune niche remodeling. CD47 silencing is especially relevant to myeloid-mediated clearance. You et al. developed engineered OMVs for co-delivery of doxorubicin and CD47-siRNA to overcome immune resistance in GBM (109). CD47 functions as a “don’t eat me” signal that suppresses phagocytosis by macrophages and microglia. Silencing CD47 may enhance tumor-cell engulfment and antigen presentation, particularly when combined with chemotherapy-induced tumor stress. This strategy directly links nanocarrier delivery, tumor-cell killing, phagocytosis checkpoint blockade, and myeloid immune activation. Beyond CD47, CD24 has emerged as another innate immune checkpoint that can suppress macrophage-mediated phagocytosis through engagement of Siglec-10 in humans or Siglec-G in mice (166). This pathway further illustrates why myeloid immune regulation should not be reduced to macrophage abundance or M1/M2-like polarization. Functional activity of an innate checkpoint depends on ligand expression, receptor-positive myeloid-cell abundance, spatial proximity between tumor and phagocytic cells, and the surrounding suppressive microenvironment. Although direct evidence for intranasal CD24-targeted nano-immunotherapy in glioma remains limited, the CD24–Siglec axis provides a useful framework for evaluating cell-specific target engagement and phagocytic competence in future myeloid-directed nanotherapies.

Overall, reversal of myeloid suppression is likely to be essential for intranasal nano-immunotherapy. Future formulations should therefore be evaluated using lineage-resolved and state-resolved myeloid profiling. At minimum, studies should distinguish resident microglia from monocyte-derived macrophages and other infiltrating myeloid populations, and then assess state-specific functions such as phagocytosis, antigen presentation, inflammatory signaling, metabolic remodeling, and immune suppression. Spatial localization should also be incorporated because identical transcriptional states may have different functional consequences when positioned in the tumor core, invasive margin, perivascular niche, or normal brain.

### 4.4 T-cell recruitment, functional state, exhaustion trajectory, and checkpoint sensitivity

T-cell dysfunction is a defining feature of GBM immune resistance, but increased CD8+ T-cell infiltration should not be equated with functional immune restoration. Contemporary T-cell biology recognizes exhaustion as a heterogeneous developmental continuum rather than a uniform dysfunctional state. CD8+ T cells may occupy progenitor/stem-like exhausted states, transitional or intermediate states, and terminally exhausted states, each with distinct proliferative capacity, persistence, effector potential, and responsiveness to checkpoint blockade (167). Consequently, a treatment that increases total intratumoral CD8+ T-cell density may still fail to generate durable antitumor immunity if the expanded population is dominated by terminally exhausted cells. Nanoparticles can support this process by silencing checkpoint molecules, modulating chemokine gradients, protecting T cells from metabolic stress, and combining immune activation with antigen release. PD-L1 silencing is one direct approach. Hsieh et al. used CXCR4-targeted nitric oxide nanoparticles to deliver PD-L1 siRNA for GBM immunotherapy (30). This strategy can reduce checkpoint-mediated inhibition while improving tumor targeting. The nitric oxide component may also influence BBB permeability and tumor vascular biology, thereby supporting delivery. By lowering PD-L1 expression in the tumor microenvironment, such platforms may increase cytotoxic T-cell activation and improve sensitivity to immune checkpoint blockade. For nano-immunotherapy, T-cell pharmacodynamics should therefore be interpreted at the state level. Progenitor/stem-like exhausted populations, commonly associated with TCF1 expression and preserved proliferative potential, may provide the cellular reservoir required for sustained responses, whereas transitional populations can represent active differentiation toward effector or exhausted fates. Terminally exhausted populations are typically characterized by more durable inhibitory-receptor expression, loss of TCF1, restricted proliferative capacity, and a more fixed dysfunctional program. These states should not be inferred from a single marker. Multiparametric flow cytometry, single-cell RNA sequencing, TCR profiling, and spatial analysis are preferable for determining whether a nanotherapy expands a renewable progenitor-like pool, promotes productive effector differentiation, or merely increases terminally exhausted infiltrates.

Engineered extracellular vesicles combined with radiation provide another route to improve checkpoint responsiveness. Tian et al. reported immune checkpoint inhibition in radiation-primed GBM using engineered extracellular vesicles (120). Radiation can increase antigen release and inflammatory signaling, thereby creating a more favorable context for checkpoint inhibition. Engineered EVs may then deliver immune-modulating signals into this primed tumor microenvironment. This approach supports the broader idea that T-cell responses in GBM often require combination strategies rather than checkpoint blockade alone. Chemokine-axis modulation can also enhance T-cell entry. Nanoparticle-mediated blockade of CXCL12/CXCR4 signaling enhanced GBM immunotherapy and enabled MRI radiomic monitoring of early responses (13). By disrupting CXCL12/CXCR4-mediated immune exclusion or suppressive trafficking, such platforms may improve T-cell infiltration and spatial access to tumor cells. This mechanism is especially relevant to glioma because immune exclusion can occur even when systemic antitumor immunity is present.

T-cell protection is another emerging concept. Lee et al. developed nitric oxide/reactive oxygen species-scavenging nanoparticles that shielded T cells and potentiated anti-PD-1 therapy in GBM (133). This strategy recognizes that the tumor microenvironment can directly damage or disable T cells through oxidative and nitrosative stress. Rather than only increasing immune activation, T-cell shielding aims to preserve effector function within hostile tumor niches. This is highly relevant to GBM, where hypoxia, oxidative stress, metabolic restriction, and myeloid-derived suppressive factors can all impair T-cell activity. Gene editing of immune-evasion factors may further enhance checkpoint sensitivity. Zou et al. showed that TME-responsive CRISPR-Cas9 nanoparticles targeting GDF15 enhanced immunotherapy efficacy in GBM (108). By disrupting a tumor-derived immunosuppressive factor, gene-editing nanomedicine may make the tumor microenvironment more permissive to T-cell attack. Similarly, OMV-mediated co-delivery of doxorubicin and CD47-siRNA can enhance immune clearance and potentially improve downstream T-cell priming by increasing phagocytosis and antigen presentation (109).

Taken together, T-cell enhancement in glioma requires more than PD-1/PD-L1 blockade. It requires a multi-step process: antigen release, dendritic-cell activation, chemokine-mediated recruitment, checkpoint relief, metabolic protection, and maintenance of cytotoxic function. Intranasal nano-immunotherapy may contribute to this process by delivering immune payloads directly to the brain tumor niche. Future studies should therefore move beyond total CD8+ T-cell infiltration and quantify the composition and trajectory of T-cell states. Relevant endpoints should include progenitor/stem-like exhausted, transitional, effector-like, and terminally exhausted populations; proliferative capacity; granzyme B and perforin expression; IFN-γ production; inhibitory-receptor co-expression; TCF1 and TOX-associated state programs; TCR clonality; and spatial proximity to tumor cells and antigen-presenting niches. Longitudinal analysis is also needed to determine whether favorable T-cell states persist after treatment or rapidly collapse into terminal exhaustion.

### 4.5 Induction of immunogenic cell death and antigen release

Immunogenic cell death is a critical mechanism linking tumor-cell killing to adaptive immunity. In GBM, therapies that kill tumor cells without generating immune activation may produce temporary tumor reduction but fail to induce durable immune control. By contrast, immunogenic cell death can release tumor antigens and damage-associated molecular patterns, including calreticulin, ATP, and HMGB1, thereby promoting dendritic-cell activation, antigen presentation, and subsequent T-cell priming (168). Nanomedicine can promote or amplify immunogenic cell death through chemotherapy, radiotherapy enhancement, photodynamic therapy, cuproptosis, oxidative stress modulation, or combined multimodal approaches (29, 169). Recent analyses of nanotechnology-mediated remodeling of the GBM tumor microenvironment further support ICD as an important interface between tumor-cell stress and immune activation (170). The bacteria-mediated metformin-loaded peptide hydrogel reported by Zhu et al. provides an example of local immune microenvironment reprogramming through combined metabolic and microbial mechanisms (130). This type of platform may create tumor stress while providing immune-stimulatory signals. Although it is not an intranasal formulation, it supports the principle that tumor-cell stress and immune activation should be coordinated rather than treated as separate therapeutic goals.

Radiation-responsive nanoplatforms are particularly relevant because radiotherapy is already part of standard GBM treatment. Wang et al. developed an engineered nanoplatform with tropism toward irradiated GBM that augmented radioimmunotherapy efficacy (131). This platform exploited radiation-induced changes in the tumor to improve nanoplatform targeting and immune efficacy. Radiotherapy can induce DNA damage, antigen release, and inflammatory signaling, while nanotherapy can amplify these effects and reduce off-target toxicity. This provides a strong rationale for combining intranasal nanomedicine with radiation-primed immune activation in future studies. Cuproptosis-mediated immune activation represents another promising mechanism. Chen et al. reported a copper-coordination driven brain-targeting nanoassembly for GBM immunotherapy through cuproptosis-mediated tumor immune microenvironment reprogramming (132). Cuproptosis may induce mitochondrial and metabolic stress, leading to tumor-cell death and immune activation. In GBM, where metabolic adaptation is closely linked to immune suppression, cuproptosis-based nanomedicine may create a bridge between metabolic vulnerability and antitumor immunity.

Photodynamic immunotherapy can also induce immunogenic tumor damage. Yan et al. developed a β-ketoenamine covalent organic framework nanoplatform combined with immune checkpoint blockade through photodynamic immunotherapy to inhibit GBM progression (140). Photodynamic therapy can generate reactive oxygen species, damage tumor cells, and promote antigen release. When combined with checkpoint blockade, this may convert local tumor destruction into systemic or durable immune responses. However, light delivery to deep brain lesions remains a major translational challenge, and future studies must address how PDT-based nanoplatforms can be clinically implemented in glioma. The OMV-based co-delivery system developed by You et al. combines chemotherapy-induced tumor stress with CD47-siRNA-mediated phagocytosis checkpoint blockade (109). This is a strong example of how antigen release and immune clearance can be coordinated. Doxorubicin can promote tumor-cell damage and antigen availability, while CD47 silencing can enhance macrophage or microglia-mediated phagocytosis (171–176). OMVs may further provide innate immune stimulation. Such multi-layered designs are likely to be more effective than single-function nanocarriers in GBM.

In summary, induction of immunogenic cell death provides the antigenic substrate for immune reprogramming. However, antigen release alone is insufficient unless it is paired with dendritic-cell activation, myeloid reprogramming, checkpoint relief, and T-cell preservation. Therefore, the most effective intranasal nano-immunotherapy systems may be those that combine tumor-cell stress with immune payload delivery. Future validation should include not only tumor volume and survival, but also markers of immunogenic cell death, antigen presentation, dendritic-cell activation, T-cell infiltration, macrophage phagocytosis, cytokine induction, and long-term immune memory. **Figure 3** illustrates the major immune processes through which nano-immunotherapy may remodel the glioma microenvironment. Particular emphasis is placed on state-resolved myeloid and T-cell responses rather than simplified M1/M2 polarization or total CD8+ T-cell infiltration alone.

**Figure 3:**
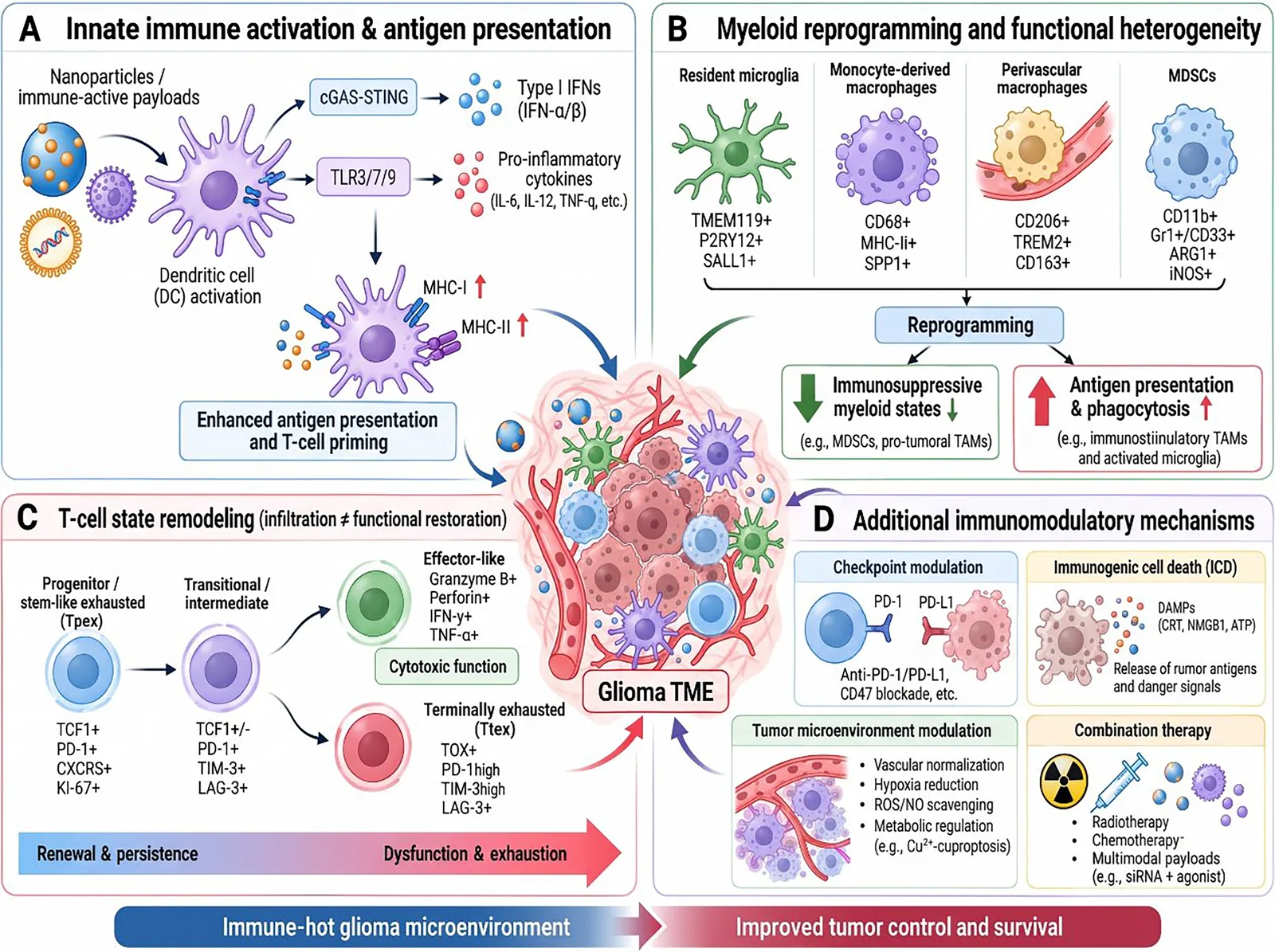
Immune remodeling mechanisms of glioma nano-immunotherapy. **(A)** Nanoplatforms activate innate immune sensing and enhance antigen presentation. **(B)** Myeloid responses are reprogrammed across resident microglia, monocyte-derived macrophages, perivascular macrophages, and MDSCs. **(C)** T-cell responses are evaluated by functional state rather than infiltration alone. **(D)** Additional mechanisms include checkpoint modulation, immunogenic cell death, tumor microenvironment modulation, and combination therapy, collectively supporting improved tumor control and survival.

## 5 Evidence hierarchy and intranasal readiness of therapeutic payloads

The therapeutic payload is a critical determinant of whether intranasal nanomedicine can move beyond brain delivery toward immune modulation. However, payloads discussed in glioma nano-immunotherapy differ substantially in their route-specific evidence. Only a limited number of payloads or payload classes have been evaluated through intranasal administration in glioma models, whereas many others are supported by systemic, local, or non-intranasal nanotherapy studies and should therefore be regarded as proposed candidates for future intranasal adaptation.

### 5.1 Level I: direct intranasal glioma nano-immunotherapy evidence

Level I evidence requires the direct experimental integration of intranasal administration, nanocarrier-mediated delivery, glioma treatment, and measurable immune modulation. Among the studies reviewed here, T7 peptide-mediated siRNA nanomicelles represent a key example because intranasal administration was directly incorporated into an immunotherapeutic glioma strategy. Such studies provide the strongest route-specific support for intranasal nano-immunotherapy. At present, direct intranasal evidence in glioma is strongest for delivery-oriented payloads and more limited for immune-specific payloads (32). Intranasal chemotherapy-loaded or temozolomide-conjugated nanoparticles have been evaluated in intracranial glioma models and provide proof-of-concept that nose-to-brain nanodelivery can produce therapeutic effects (177, 178). However, these studies mainly support the delivery feasibility of intranasal nanomedicine rather than immune-specific reprogramming (179). For immune-related payloads, T7 peptide-mediated siRNA nanomicelles provide one of the most directly relevant examples because this platform combines intranasal administration, nanomicelle-mediated nucleic-acid protection, brain tumor targeting, and glioma immunotherapy (32). This study supports the feasibility of intranasal nucleic-acid delivery in glioma, but it should not be generalized to all siRNA, CRISPR, checkpoint, or innate immune agonist payloads (46, 180). Future studies should determine whether intranasal siRNA platforms can reproducibly achieve tumor-localized target knockdown, immune pharmacodynamic activity, survival benefit, and acceptable nasal-brain safety.

### 5.2 Level II: intranasal glioma nanodelivery without demonstrated immune reprogramming

Level II evidence comprises studies in which nanocarriers were administered intranasally and achieved brain or glioma delivery, tumor suppression, or survival benefit, but immune microenvironment reprogramming was not directly demonstrated. Intranasal temozolomide-loaded or chemotherapy-oriented nanoparticles, ferritin-based nose-to-brain nanovectors, and related delivery platforms therefore provide proof-of-concept for route feasibility rather than direct evidence of nano-immunotherapy (17, 22, 25, 26). These studies are important because they validate intranasal access to intracranial glioma, but their therapeutic effects should not be interpreted as evidence of immune modulation unless immune pharmacodynamic endpoints are measured.

### 5.3 Level III: non-intranasal nano-immunotherapy supporting future intranasal adaptation

Level III evidence includes glioma nano-immunotherapy studies that demonstrate relevant immune mechanisms or payload efficacy through systemic, local, or other non-intranasal routes. These studies provide mechanistic justification for future intranasal adaptation but do not establish that equivalent delivery, cellular uptake, target engagement, or immune pharmacodynamics can be achieved after nasal administration. Representative examples include non-intranasal STING/TLR3-oriented nanoplatforms (107, 132), PD-L1 siRNA delivery systems (30), CD47-siRNA-based phagocytosis-checkpoint modulation (109), GDF15-targeted CRISPR-Cas9 nanoparticles (108), CXCL12/CXCR4-directed nanotherapy (13), engineered extracellular vesicles (181), and multimodal immunogenic-cell-death platforms (113). These studies therefore provide mechanism- and payload-level support for future intranasal adaptation rather than direct evidence of intranasal efficacy. These include cGAS-STING or TLR3 agonists, PD-L1 siRNA, CD47-siRNA, GDF15-targeted CRISPR-Cas9 systems, CXCL12/CXCR4 inhibitors, engineered extracellular vesicles used in radiation-primed tumors, and multimodal payloads designed to induce immunogenic cell death or protect T cells (30, 107). These payloads should be described as candidates for future intranasal adaptation rather than as established intranasal payloads. Their relevance lies in the immune barriers they address. STING or TLR agonists may be suitable when the main problem is weak innate priming (101, 107). PD-L1 siRNA may be useful when T cells are present but inhibited by checkpoint signaling (30). CD47-siRNA may be more relevant in myeloid-rich tumors where phagocytosis is suppressed (109). CXCL12/CXCR4 inhibitors may be useful for immune-excluded or perivascular suppressive niches (13, 31). Engineered EVs and radiation-primed systems may be most rational when antigen release and checkpoint responsiveness need to be coordinated (181). However, none of these mechanisms should be assumed to occur after nasal administration unless tumor delivery, cell-specific uptake, target modulation, and immune pharmacodynamics are directly demonstrated.

### 5.4 Payload selection according to immune barrier and intranasal feasibility

Payload selection for future intranasal nano-immunotherapy should be mechanism-guided and evidence-ranked. If the goal is to initiate immune priming, innate agonists such as STING or TLR agonists may be rational, but they carry a high risk of nonspecific neuroinflammation and require strict regional cytokine mapping (18, 101). If the goal is to silence immune-evasion pathways, siRNA or CRISPR payloads may provide target specificity, but their success depends on cytosolic delivery, tumor-localized knockdown or editing, and avoidance of normal-brain exposure (46, 108). If the goal is to reverse myeloid suppression, CD47-siRNA, CXCL12/CXCR4 inhibitors, or macrophage-reprogramming agents may be appropriate, but they require cell-specific validation in macrophages, microglia, and perivascular immune niches (31, 106, 109). If the goal is to provide antigenic substrate, chemotherapy-, radiation-, photodynamic-, or cuproptosis-associated payloads may be useful, but they should be paired with evidence of antigen presentation and T-cell activation rather than tumor-cell killing alone (182–184). Thus, the most suitable payload is not necessarily the most immunostimulatory or mechanistically complex one. It is the payload that can be delivered reproducibly through the nasal route, reach glioma-relevant compartments, engage the intended immune pathway, improve antitumor efficacy, and maintain an acceptable mucosal, neurological, and systemic safety profile (32, 46). **Supplementary Table S2** summarizes representative nano-immunotherapeutic strategies for glioma immune microenvironment reprogramming. **Figure 4** distinguishes payload classes according to both immune mechanism and route-specific evidence strength. This framework prevents mechanistic findings from systemic or local nanotherapy studies from being interpreted as established intranasal efficacy.

**Figure 4:**
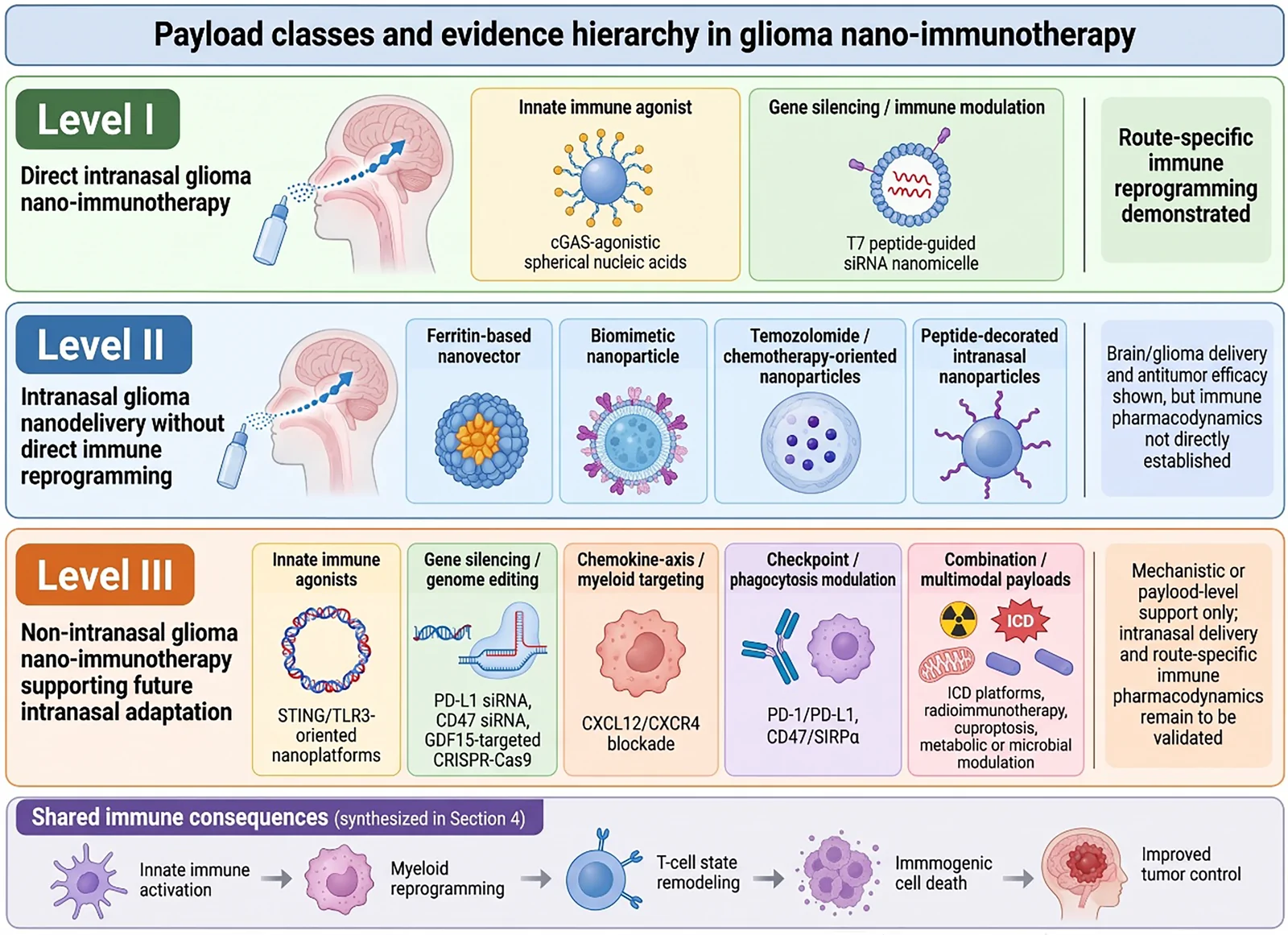
Payload classes and evidence hierarchy for glioma nano-immunotherapy. Therapeutic payloads are stratified into Level I direct intranasal glioma nano-immunotherapy, Level II intranasal glioma nanodelivery without demonstrated immune reprogramming, and Level III non-intranasal nano-immunotherapy supporting future intranasal adaptation. Major payload classes include innate immune agonists, gene-silencing or genome-editing agents, chemokine-axis modulators, checkpoint-directed strategies, and multimodal immunogenic therapies.

## 6 Preclinical validation: how to prove intranasal nano-immunotherapy works

Preclinical validation is essential for translating intranasal nano-immunotherapy from an attractive concept into a clinically credible strategy (185, 186). Many nanomedicine studies show promising tumor inhibition, but insufficient attention is often paid to delivery mechanism, biodistribution, immune profiling, reproducibility, and safety (187). For glioma, validation must be especially rigorous because the therapeutic system must overcome nasal barriers, reach intracranial tumor tissue, interact with the brain immune microenvironment, and avoid neurotoxicity (53, 145). Therefore, preclinical evaluation should proceed through four validation layers: formulation quality control, including physicochemical characterization and *in vitro* functional assays; delivery validation, including nasal performance and nose-to-brain biodistribution; therapeutic validation, including orthotopic glioma efficacy, immune profiling, survival analysis, and recurrence assessment; and safety validation, including local, neurological, and systemic toxicity testing (188). To improve translational reliability, intranasal glioma nano-immunotherapy should be evaluated through an integrated validation pipeline that includes formulation quality, nasal performance, biodistribution, orthotopic efficacy, immune pharmacodynamics, survival benefit, and safety. A proposed preclinical validation checklist is provided in **Supplementary Table S3**.

### 6.1 *In vitro* validation

*In vitro* validation begins with physicochemical characterization of the nanoplatform. Essential parameters include particle size, polydispersity index, zeta potential, morphology, stability, drug-loading efficiency, encapsulation efficiency, and release kinetics. These parameters are not merely technical details; they directly influence nasal retention, mucus penetration, epithelial transport, cellular uptake, immune activation, and toxicity. For intranasal delivery, additional assays should evaluate mucoadhesion, mucus diffusion, enzymatic stability in nasal secretions, and compatibility with nasal epithelial cells. For nucleic acid-loaded systems, payload protection and functional release are particularly important. Tang et al. showed that T7 peptide-mediated nanomicelles could support siRNA delivery through intranasal administration for glioma immunotherapy (32). Such platforms should be validated for siRNA stability, protection from RNase degradation, cellular uptake, endosomal escape, and target-gene knockdown. Similar principles apply to PD-L1 siRNA nanoparticles, CRISPR-Cas9 nanoparticles targeting GDF15, and OMV-based CD47-siRNA co-delivery systems (30, 108, 109).

For immunostimulatory nanoplatforms, *in vitro* validation should include immune activation markers rather than only tumor-cell cytotoxicity. Polyinosinic/polycytidylic lipid nanoparticles enhanced immune-cell infiltration and survival in GBM models, but their preclinical evaluation should also include TLR3 pathway activation, interferon-related gene expression, dendritic-cell maturation, cytokine secretion, and immune-cell viability (107). Similarly, cGAS/STING-oriented systems should be tested for STING activation, type I interferon production, chemokine induction, and inflammatory toxicity. For biomimetic and intranasal platforms, cellular models should include more than glioma cell lines. Ferreira et al. developed biomimetic nanoparticles for nose-to-brain GBM-targeted therapy, highlighting the need to evaluate interactions with nasal epithelial cells, brain endothelial cells, glioma cells, macrophages, microglia, dendritic cells, and T cells (17). Co-culture systems, spheroids, organoids, and microfluidic nasal-brain barrier models may provide more informative data than conventional two-dimensional tumor-cell assays. Important endpoints include epithelial permeability, mucus transport, tumor-cell uptake, immune-cell activation, phagocytosis, T-cell killing, and cytokine release. *In vitro* safety testing should also be included early. Nanoparticles intended for intranasal administration should be tested for nasal epithelial toxicity, ciliary function impairment, barrier disruption, inflammatory cytokine induction, hemolysis, complement activation, and effects on neuronal or glial cells. These assays cannot replace animal safety studies, but they can identify formulation risks before *in vivo* testing.

### 6.2 Nose-to-brain biodistribution and pharmacokinetics

Biodistribution is one of the most important validation domains for intranasal nano-immunotherapy. Demonstrating therapeutic efficacy alone is not sufficient; studies must show that the formulation reaches the brain and accumulates in glioma-relevant regions (95). Where quantitative data are available, biodistribution should be interpreted together with the administered dose and therapeutic outcome. A useful pharmacological chain is: administered intranasal dose → fraction retained in the nasal cavity → fraction reaching the brain → tumor-selective exposure → cellular target engagement → pharmacodynamic and therapeutic effect. Increased whole-brain accumulation alone should not be considered evidence of an effective glioma dose, because a substantial fraction of the formulation may remain in the olfactory bulb, normal brain parenchyma, cerebrospinal-fluid-associated spaces, or other non-tumor compartments. Therefore, studies should report absolute or normalized tumor exposure, tumor-to-normal brain ratios, tumor-to-blood ratios, exposure duration, and, when feasible, the relationship between these parameters and tumor inhibition, survival, or immune pharmacodynamic responses. Key endpoints should include nasal cavity retention, olfactory bulb accumulation, distribution across different brain regions, tumor-to-normal brain ratio, blood exposure, liver and spleen accumulation, kidney clearance, and duration of intracranial retention (44). Fluorescence imaging, radiolabeling, mass spectrometry, quantitative PCR, MRI, PET, or ex vivo tissue quantification may be used depending on the payload and carrier (20). Intranasal glioma nanomedicine studies provide useful examples, including ferritin-based nanovectors, biomimetic nanoparticles, and peptide-decorated nanocarriers that have directly assessed brain or tumor-associated delivery after nasal administration (17, 22, 97). Sandbhor et al. demonstrated that targeted intranasal nanodelivery of chemotherapy suppressed intracranial GBM growth and prolonged survival (25). Marrocco et al. provided a particularly informative dose–exposure–response example using the ferritin-based nanovector The-0504 carrying Genz-644282. In orthotopic GL261 glioma-bearing mice, intranasal The-0504 was administered every two days at 0.3 or 0.9 mg/kg, expressed as Genz-644282, whereas the intravenous comparison dose was 2.0 mg/kg. After six intranasal administrations, mean tumor volumes were reduced from 5.4 ± 1.2 mm³ in the empty-carrier control group to 1.5 ± 0.5 mm³ and 1.4 ± 0.3 mm³ at 0.3 and 0.9 mg/kg, respectively. At 0.9 mg/kg, quantitative drug analysis further demonstrated preferential exposure in the tumor-bearing hemisphere with negligible plasma levels, supporting tumor-directed delivery rather than systemic redistribution. Importantly, the similar tumor-volume reduction observed at 0.3 and 0.9 mg/kg also suggests that increasing the administered dose did not proportionally increase the measured antitumor effect under these experimental conditions, highlighting the importance of defining an optimal biological dose rather than assuming a linear dose–response relationship (22). Ferreira et al. developed biomimetic nanoparticles for nose-to-brain GBM-targeted therapy (17). Koh et al. further demonstrated that intranasal temozolomide-conjugated superparamagnetic iron oxide nanoparticles combined with transcranial magnetic stimulation prolonged survival in U87 xenograft models while using less than one-tenth of the conventional temozolomide dose. The treated mice survived approximately twice as long as untreated controls, suggesting that improved spatial delivery may partially compensate for a lower administered dose. However, because tumor-site drug concentrations were not quantitatively linked to survival in this study, the results demonstrate dose-sparing therapeutic efficacy rather than a fully defined tumor exposure–response relationship (26). These studies collectively support the feasibility of intranasal delivery, but they also show why detailed biodistribution analysis is essential. Biodistribution should be evaluated in a tiered manner. The first tier is brain exposure, including olfactory bulb, anterior brain, deep brain, and whole-brain accumulation. The second tier is tumor-selective exposure, including tumor-to-normal brain ratio, invasive-margin accumulation, and tumor-to-blood ratio. The third tier is immune-cell-relevant delivery, including payload uptake or pharmacodynamic activity in glioma cells, macrophages, microglia, dendritic cells, endothelial-associated immune niches, or T cells. This tiered evaluation is essential because total brain accumulation may overestimate therapeutic delivery if the formulation does not reach the tumor or the intended immune-cell compartment.

For immune payloads, biodistribution should be linked to pharmacodynamics. For example, if cGAS-agonistic spherical nucleic acids are used to reprogram the GBM immune microenvironment, studies should determine whether the immune activation occurs primarily in tumor tissue, normal brain, nasal mucosa, cervical lymph nodes, or peripheral organs. If siRNA nanomicelles are administered intranasally, validation should link the administered siRNA dose to quantitative tumor accumulation, target-gene knockdown, tumor inhibition, and survival rather than reporting brain fluorescence or distribution alone (32). This distinction is critical because immune activation in the wrong compartment may cause toxicity without improving antitumor efficacy. Direct route-to-route comparisons are particularly informative. Marrocco et al. compared intranasal and intravenous administration of the same ferritin-based The-0504 nanovector in orthotopic GL261 glioma-bearing mice (22). After six treatments, intranasal administration at 0.3 mg/kg reduced tumor volume by more than 70%, whereas intravenous administration at 2.0 mg/kg produced an approximately 53% reduction, indicating greater antitumor activity with approximately sevenfold less administered drug through the intranasal route. Following intranasal administration at 0.9 mg/kg, the amount of Genz-644282 in the tumor-bearing hemisphere was approximately fivefold higher than that in the contralateral hemisphere, while plasma concentrations remained below approximately 11 ng/g blood during the evaluated period. These findings provide quantitative evidence of tumor-selective exposure and dose-sparing efficacy after intranasal administration. However, because directly matched intravenous plasma exposure and tumor-to-normal-brain pharmacokinetic parameters were not reported, the study does not establish a complete quantitative systemic-exposure advantage of the intranasal route. This distinction illustrates why route superiority should be evaluated using matched pharmacokinetic, biodistribution, efficacy, and toxicity endpoints rather than brain accumulation alone. A separate same-dose pharmacokinetic comparison of paclitaxel-loaded PLGA nanoparticles also illustrates that the advantage of intranasal administration is not necessarily higher total brain exposure. At 5 mg/kg, intranasal administration produced lower plasma, liver, and kidney exposure than intravenous administration, whereas total brain Cmax and AUC were not higher after intranasal dosing. Specifically, plasma Cmax decreased from 10.13 to 1.82 ng/mL, liver AUC0–24 from 274.9 to 113.5 ng·h/mL, and kidney AUC0–24 from 141.3 to 22.0 ng·h/mL, while brain AUC0–24 was 2.0 ng·h/mL after intranasal administration compared with 4.5 ng·h/mL after intravenous administration (189). These data emphasize that the pharmacological value of intranasal delivery may lie in altered tissue partitioning and reduced peripheral exposure rather than universally increased whole-brain exposure.

Pharmacokinetic studies should therefore include matched intranasal-versus-systemic comparisons whenever feasible. Relevant endpoints should include administered dose, brain and tumor AUC/Cmax, tumor-to-normal-brain and tumor-to-blood ratios, plasma exposure, liver and spleen distribution, renal clearance, and route-specific toxicity. Only when these parameters are considered together with target engagement and therapeutic outcome can a meaningful pharmacological advantage of intranasal administration be established. For clinical translation, dose scaling from rodents to humans remains a major challenge because nasal anatomy, olfactory epithelial surface area, mucociliary clearance, and administration volume differ substantially between species. Accordingly, future intranasal glioma nanotherapy studies should report, whenever technically feasible, the administered dose, dosing volume and frequency, fraction reaching the brain, quantitative tumor exposure, tumor-to-normal brain ratio, systemic exposure, and duration of tumor retention. These pharmacokinetic parameters should then be related to pharmacodynamic endpoints and therapeutic outcomes. Detectable brain accumulation should be regarded as evidence of transport, whereas therapeutically meaningful delivery requires demonstration that sufficient payload reaches the glioma site, engages the intended molecular or cellular target, and produces a reproducible biological or survival benefit. Dose escalation should therefore be interpreted through tumor exposure and pharmacodynamic saturation rather than administered dose alone.

### 6.3 Orthotopic glioma models

Model selection is critical for evaluating intranasal nano-immunotherapy. Subcutaneous glioma models are convenient for measuring tumor volume and testing basic antitumor activity, but they do not reproduce the BBB/BTB, brain extracellular matrix, intracranial pressure, neuroimmune environment, or invasive growth pattern of glioma. Therefore, orthotopic models are essential for validating any brain-targeted or immune-modulating therapy. Intracranial GL261, CT-2A, U87-luc, patient-derived xenograft, and genetically engineered models each provide different advantages and limitations (190–192). For immunotherapy studies, immunocompetent models such as GL261 or CT-2A are especially important because they preserve host immune interactions. Many immune mechanisms, including T-cell infiltration, macrophage/microglia reprogramming, dendritic-cell activation, and checkpoint sensitivity, cannot be adequately evaluated in immunodeficient xenograft models. Studies such as cGAS-agonistic SNA-mediated immune reprogramming, biomimetic nanoparticle-mediated TIME remodeling, polyinosinic/polycytidylic lipid nanoparticle therapy, cuproptosis-mediated nano-immunotherapy, T-cell shielding nanoparticles combined with anti-PD-1, and OMV-based CD47-siRNA co-delivery illustrate the importance of immune-competent preclinical validation (73, 107, 133).

Intranasal delivery studies should also use orthotopic models because the route of administration depends on anatomical transport to the brain. Sandbhor et al. used an intracranial mouse model to evaluate intranasal nanodelivery of chemotherapy (25). Marrocco et al. evaluated ferritin-based nose-to-brain nanovectors in glioma-bearing models (22). Tang et al. used intranasal administration of T7 peptide-mediated siRNA nanomicelles for glioma immunotherapy (32). These studies are important because they connect nasal delivery with intracranial disease rather than relying on ectopic tumor models. Model selection should be aligned with the question being asked. If the goal is to test nose-to-brain transport, orthotopic models with imaging-traceable tumors may be sufficient. If the goal is to test immunotherapy, immunocompetent syngeneic models are necessary. If the goal is to test human tumor heterogeneity, patient-derived models may be informative but have limited immune relevance when implanted into immunodeficient mice. Ideally, future studies should use a staged validation strategy: first demonstrate delivery and basic efficacy, then confirm immune mechanisms in immunocompetent orthotopic models, and finally validate selected findings in patient-derived or humanized models. For advancement toward translational development, tumor-growth inhibition in subcutaneous or immunodeficient xenograft models should not be considered sufficient. A candidate immune-active intranasal nanoplatform should demonstrate reproducible therapeutic benefit, including survival improvement, in at least an immunocompetent orthotopic setting in which the relevant host immune interactions are preserved.

### 6.4 Immune profiling endpoints

Immune profiling is essential because nano-immunotherapy aims to remodel the tumor immune microenvironment, not merely reduce tumor size. Key immune endpoints should include innate immune activation, myeloid-cell state, dendritic-cell maturation, T-cell infiltration, checkpoint expression, cytokine production, antigen presentation, and spatial immune organization. Without immune profiling, it is difficult to distinguish true immune reprogramming from nonspecific tumor toxicity. Flow cytometry can quantify immune-cell populations such as CD8+ T cells, CD4+ T cells, regulatory T cells, dendritic cells, macrophages, microglia, MDSCs, and neutrophil-like cells (193–197). Immunofluorescence and immunohistochemistry can show spatial localization of CD8, CD4, Foxp3, Iba1, CD11b, CD68, CD206, MHC-II, PD-L1, granzyme B, and cleaved caspase markers. Cytokine profiling can evaluate interferon-β, interferon-γ, TNF-α, IL-6, CXCL10, CCL2, and other inflammatory mediators. For innate immune agonist platforms such as cGAS-agonistic SNAs, STING-reprogramming systems, and polyinosinic/polycytidylic lipid nanoparticles, interferon-stimulated genes and antigen-presentation markers are especially important (101, 107).

Myeloid profiling should be a major focus because GBM is characterized by profound myeloid heterogeneity (154). Biomimetic nanoparticles that remodel the immunosuppressive microenvironment, CXCR4/CXCL12-targeted synthetic protein nanoparticles, nanoparticle-mediated CXCL12/CXCR4 blockade, cuproptosis-mediated immune reprogramming, and OMV-based CD47-siRNA co-delivery all require lineage- and state-resolved evaluation of myeloid responses (13, 31, 109, 132, 198). Importantly, myeloid pharmacodynamics should move beyond the conventional M1/M2-like framework. Resident microglia, monocyte-derived macrophages, perivascular macrophages, and other infiltrating myeloid populations occupy distinct anatomical niches and can adopt heterogeneous transcriptional, metabolic, phagocytic, antigen-presenting, and immunosuppressive states (151). Markers such as TMEM119, P2RY12, and SALL1 can help identify resident microglial populations, whereas MHC-II, CD68, SPP1, TREM2, CD163, CD206, and phagocytosis-related molecules can contribute to broader state characterization. Conventional markers such as CD86, iNOS, Arg1, CD206, and CD163 may remain informative as components of multiparametric panels, but they should not be used alone to assign binary M1/M2 identities.

T-cell profiling should similarly distinguish immune-cell abundance from functional immune restoration. Increased CD8+ T-cell infiltration does not necessarily indicate an effective antitumor response because intratumoral CD8+ T cells may occupy progenitor/stem-like exhausted, transitional/intermediate, effector-like, or terminally exhausted states (199). These populations differ in proliferative capacity, persistence, cytotoxic function, and responsiveness to checkpoint blockade. Accordingly, PD-L1 siRNA nanoparticles, T-cell-shielding nanoparticles that potentiate anti-PD-1 therapy, CRISPR-Cas9 nanoparticles targeting GDF15, and OMV/CD47-siRNA systems should be evaluated not only for total CD8+ T-cell infiltration but also for T-cell-state composition and functional transitions (30, 108, 109, 133). Multiparametric assessment of TCF1, Ki-67, PD-1, TIM-3, LAG-3, TOX, granzyme B, perforin, and IFN-γ may help distinguish renewable progenitor-like populations from productive effector responses and terminally exhausted states (200).

More broadly, immune pharmacodynamic validation should incorporate four complementary dimensions: cell-specific target engagement, functional immune-state changes, spatial localization, and temporal persistence. Single-cell RNA sequencing can resolve lineage-specific immune states and ligand–receptor interactions, whereas spatial transcriptomics and multiplex imaging can determine whether functionally relevant T cells or myeloid populations localize to tumor cores, invasive margins, perivascular niches, or regions of direct tumor-cell contact. Spatial and temporal persistence are equally important. Recent observations in GBM indicate that durable antitumor responses can be associated with persistent T-cell infiltration, local expansion of tumor-reactive populations, and sustained spatial proximity between T cells and tumor cells even after the initiating therapeutic stimulus has diminished (201). Therefore, immune pharmacodynamic assessment should include repeated or longitudinal sampling whenever feasible and should distinguish transient cytokine induction from persistent tumor–immune engagement. Spatial immune–tumor proximity may provide a more functionally informative endpoint than bulk immune-cell density alone. TCR sequencing can further assess clonal expansion and persistence of tumor-reactive T-cell populations. Longitudinal analyses are particularly important because a transient increase in cytokines or immune-cell abundance may not represent durable immune reprogramming. For intranasal nano-immunotherapy, a convincing pharmacodynamic chain should therefore demonstrate nanoplatform delivery to the glioma, uptake by the intended cellular compartment, molecular target engagement, transition toward the predicted functional immune state, appropriate spatial tumor–immune interaction, and persistence of these changes over time. Immune-cell abundance or cytokine alterations alone should not be considered sufficient evidence of functional immune restoration.

### 6.5 Survival, recurrence, and safety endpoints

Tumor volume reduction is an important but insufficient endpoint for glioma nano-immunotherapy. GBM is lethal because of recurrence, invasion, and treatment resistance. Therefore, survival extension, delayed recurrence, neurological function, and safety should be prioritized. Studies should report median survival, long-term survivors, tumor recurrence patterns, and, when possible, rechallenge experiments to assess immune memory. Imaging-based tumor monitoring should be complemented by histological and immune analyses. Intranasal delivery studies have already shown the importance of survival endpoints. Sandbhor et al. reported that intranasal targeted nanodelivery of chemotherapy suppressed GBM growth and prolonged survival in an intracranial model (25). Marrocco et al. showed that ferritin-based nose-to-brain nanovectors reduced tumor growth and improved survival (22). Koh et al. reported survival improvement after intranasal temozolomide-conjugated nanoparticles combined with transcranial magnetic stimulation (26). These studies support survival as a central endpoint for nose-to-brain glioma nanomedicine.

For immune-focused platforms, survival should be interpreted alongside immune activation and safety. Polyinosinic/polycytidylic lipid nanoparticles improved survival and enhanced immune-cell infiltration in a GBM mouse model (107). T-cell shielding nanoparticles potentiated anti-PD-1 therapy (133). OMV-based doxorubicin and CD47-siRNA co-delivery potentiated GBM immunotherapy (109). These studies illustrate that survival benefit is most informative when connected to mechanistic evidence, such as increased T-cell function, myeloid reprogramming, checkpoint modulation, or phagocytosis. Safety evaluation is particularly important for intranasal nano-immunotherapy. Local nasal toxicity should be assessed by histology of the nasal mucosa, ciliary integrity, epithelial inflammation, mucus production, olfactory epithelium damage, and behavioral tests related to smell when feasible. Brain safety should include neuroinflammation, edema, neuronal injury, microglial overactivation, demyelination, seizure-like behavior, and neurological function. Systemic safety should include body weight, hematology, serum biochemistry, liver and kidney histology, cytokine-release profiles, and immune-related adverse events.

The safety requirements are even higher for potent immune agonists, bacterial vesicles, CRISPR systems, and oxidative stress-modulating platforms. STING/TLR activation may cause excessive inflammation; OMV-based systems may cause endotoxin-related toxicity; CRISPR systems may cause off-target editing; and metal-based or ROS-generating platforms may damage normal brain tissue (202). Therefore, future studies should include dose-escalation, repeated-dose toxicity, biodistribution persistence, inflammatory recovery, and long-term neurological monitoring. Finally, recurrence and immune memory should become more common endpoints. If nano-immunotherapy successfully converts glioma into an immune-responsive tumor, it may not only shrink established tumors but also delay recurrence or generate protective memory. Rechallenge experiments, memory T-cell analysis, and long-term survivor profiling could help determine whether treatment produces durable immune surveillance. This is especially important for GBM, where short-term tumor inhibition often fails to translate into lasting control. A rigorous preclinical validation pipeline should therefore connect formulation quality, brain delivery, immune remodeling, survival benefit, recurrence control, and safety into one integrated framework. **Figure 5** outlines the key validation steps required to prove that intranasal nano-immunotherapy achieves brain delivery, glioma targeting, immune remodeling, and therapeutic benefit. It emphasizes that survival improvement and safety assessment should be linked with biodistribution and immune pharmacodynamic evidence.

**Figure 5:**
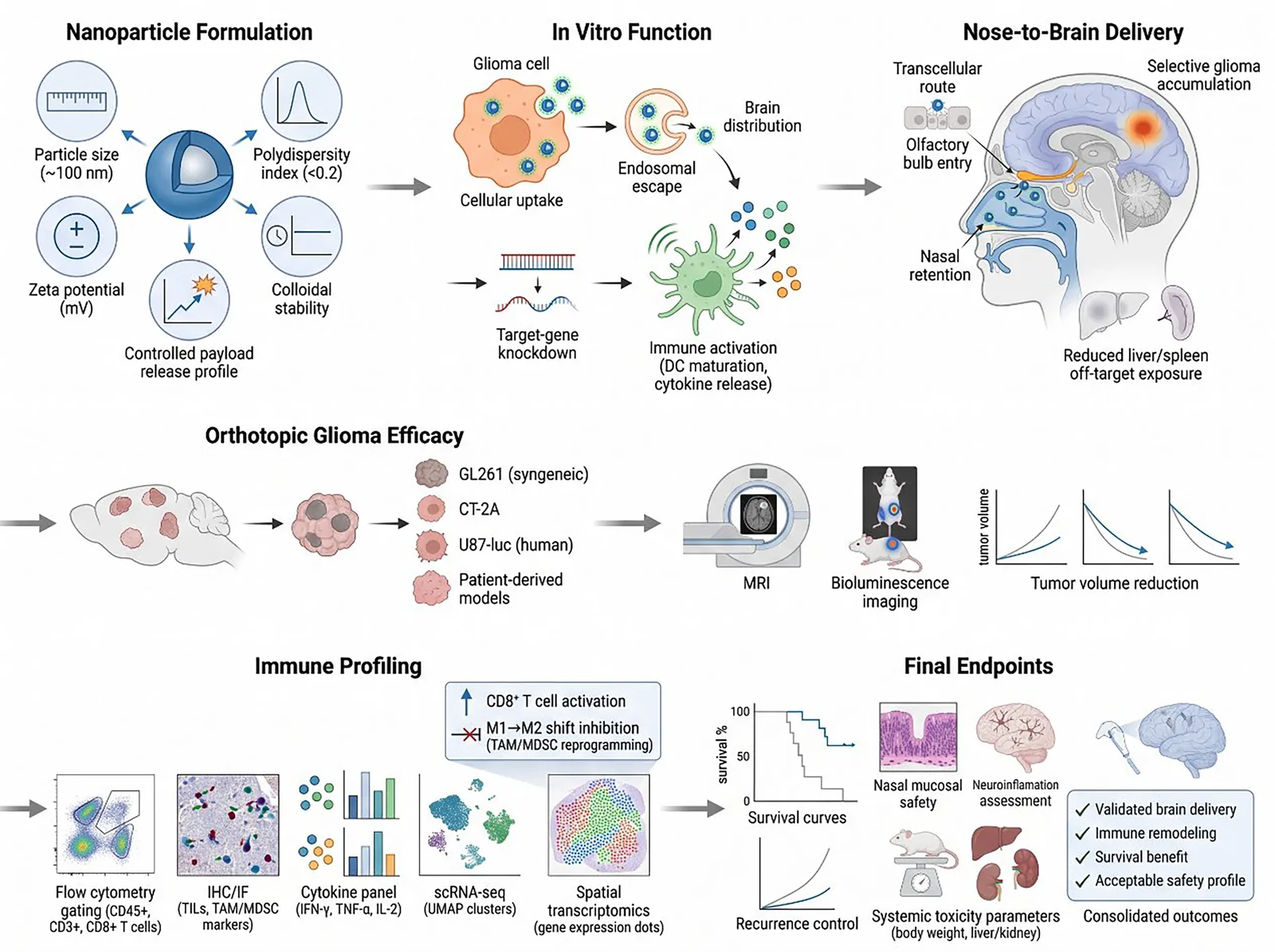
Preclinical validation pipeline for intranasal glioma nano-immunotherapy. A rigorous workflow should integrate nanoparticle characterization, *in vitro* functional testing, nasal performance, nose-to-brain biodistribution, orthotopic glioma efficacy, immune pharmacodynamic profiling, survival and recurrence assessment, and local, neurological, and systemic safety evaluation to support translational development.

## 7 Translational challenges and clinical bridging

Although intranasal nano-immunotherapy offers an attractive strategy for glioma treatment, its clinical translation remains challenging. The preclinical evidence supporting nose-to-brain nanomedicine and nano-immunotherapy is growing, but most studies are still performed in small-animal models with controlled dosing, simplified anatomy, and limited long-term safety evaluation (44, 95). Translation to patients requires more than showing tumor inhibition in mice. It requires reproducible formulation manufacturing, quantitative delivery assessment, immune pharmacodynamic monitoring, safety evaluation in relevant anatomical compartments, and clinically usable biomarkers (203, 204). Recent analyses of intranasal nanomaterial-based GBM therapy further emphasize that reproducible nasal deposition, clinically relevant dosing, formulation standardization, and regulatory-compatible development remain major barriers between preclinical efficacy and human application (205). Therefore, the next stage of development should focus on bridging the gap between experimental feasibility and clinical applicability.

### 7.1 Current clinical evidence and the translational gap

Although numerous intranasal nanoformulations have shown therapeutic activity in cellular and rodent glioma models, no completed phase I/II clinical trial has yet directly evaluated an intranasal nanoformulation-based immunotherapy for glioma. Therefore, the current clinical evidence should be interpreted through two related but distinct categories: human intranasal glioma therapy using non-nanoparticle formulations, and human glioma nanomedicine using non-intranasal administration routes (206). The most relevant human intranasal evidence comes from perillyl alcohol and its highly purified formulation NEO100 (207). Early Brazilian phase I/II studies explored repeated intranasal perillyl alcohol administration in adults with recurrent malignant gliomas and reported acceptable tolerability with signals of clinical activity. In the United States, NEO100 was evaluated in an open-label phase I/IIa trial in recurrent GBM. In the completed phase I part of this study, 12 patients received intranasal NEO100 four times daily using a nebulizer and nasal mask. Four dose levels were tested: 96 mg per dose, 144 mg per dose, 192 mg per dose, and 288 mg per dose, corresponding to total daily doses of 384, 576, 768, and 1152 mg/day, respectively. Treatment cycles lasted 28 days. Intranasal NEO100 was well tolerated at all dose levels, with no severe treatment-related adverse events reported. Mild local or administration-related events included nasal soreness, nasal pruritus, rhinorrhea, nasal dryness, nasal discomfort, skin irritation around the nose, cough, and headache (206, 208).

However, these clinical data cannot be considered direct evidence for intranasal nano-immunotherapy. Perillyl alcohol/NEO100 is a small-molecule monoterpene rather than a nanocarrier-based immune payload (206). Conversely, several nanoparticle-based glioma therapies have entered clinical testing, but they use non-intranasal routes. For example, AGuIX gadolinium-based theranostic nanoparticles are being evaluated with radiotherapy and temozolomide in newly diagnosed GBM, but they are administered intravenously (209, 210). NanoTherm therapy uses magnetic iron-oxide nanoparticles for local thermotherapy in recurrent GBM, but this approach relies on intratumoral, intracavitary, or local nanoparticle placement rather than nose-to-brain delivery (211–213). These studies indicate that both intranasal glioma therapy and glioma nanomedicine have reached early clinical development, but the intersection of these two fields remains clinically unvalidated. Thus, a major translational gap is the absence of human studies that simultaneously integrate intranasal administration, nanocarrier design, glioma targeting, and immune microenvironment reprogramming. Representative clinical and translational evidence relevant to intranasal glioma therapy and glioma nanomedicine is summarized in **Supplementary Table S4**. Because no completed phase I/II trial has directly tested intranasal nanoformulation-based immunotherapy for glioma, **Supplementary Table S4** distinguishes direct intranasal non-nanoparticle evidence from non-intranasal nanoparticle-based glioma trials.

### 7.2 Rodent–human nasal anatomical disparities and dose translation

Dose translation is one of the most important unresolved issues in intranasal nanoformulations because rodent nose-to-brain delivery does not directly predict human nasal deposition or brain exposure (214). Rodents have a proportionally much larger olfactory epithelial region than humans, and this anatomical difference may overestimate direct nose-to-brain delivery efficiency in mouse or rat studies (215, 216). In rats and mice, the olfactory epithelium may account for approximately 40–50% of the nasal surface area, whereas in humans it generally represents less than 10% of the nasal cavity (19). In addition, rodent studies often use small-volume pipette dosing under anesthesia or restraint, which differs substantially from human self-administration using sprays, nebulizers, nasal masks, or device-assisted deposition (215, 217). Human nasal delivery is affected by nasal vestibule size, turbinate structure, airflow pattern, mucus thickness, mucociliary clearance, head position, droplet size, spray plume geometry, formulation viscosity, and interindividual nasal anatomy (52, 217–220). Therefore, a dose that achieves strong olfactory deposition and brain accumulation in rodents may not produce equivalent brain exposure in patients].

Recent intranasal glioma nanomedicine studies provide important proof-of-concept evidence but also highlight the need for quantitative dose optimization. Sandbhor et al. showed that targeted intranasal nano-delivery of chemotherapy suppressed intracranial GBM growth and prolonged survival in a mouse model (25). Marrocco et al. demonstrated that ferritin-based nanovectors enabled nose-to-brain selective delivery to glioma and improved survival (22). Ferreira et al. demonstrated brain accumulation after intranasal administration of biomimetic TMZ-loaded nanoparticles, but the available evidence primarily establishes route feasibility and brain exposure rather than a quantitative tumor dose–response relationship (17). Koh et al. reported that intranasal temozolomide-conjugated nanoparticles combined with transcranial magnetic stimulation improved survival in GBM xenograft models (26). These studies support the feasibility of intranasal delivery, but clinical translation will require standardized reporting of administered dose, retained nasal dose, brain-delivered fraction, tumor-to-normal brain ratio, systemic exposure, and dosing frequency. Whenever a systemic comparator is available, these parameters should be reported for both routes under matched experimental conditions, because differences in administered dose, formulation, dosing frequency, or sampling time can otherwise produce misleading claims of intranasal superiority. Importantly, dose translation should be based on tumor-relevant exposure and pharmacodynamic activity rather than simple body-weight scaling of the administered intranasal dose, because equivalent mg/kg doses may produce very different nasal deposition, brain penetration, and glioma exposure across species. Formulation properties further complicate dosing. Highly mucoadhesive formulations may remain longer in the nasal cavity but may also become trapped in mucus and cleared. Smaller nanoparticles may diffuse more efficiently but may show weaker retention. Positively charged particles may interact with mucins and epithelial membranes but can increase local toxicity. Viscous gels may improve residence time but reduce distribution across the nasal cavity. Therefore, the optimal formulation is unlikely to be defined by one parameter alone. Instead, dose optimization should integrate nasal deposition, mucus penetration, epithelial transport, brain distribution, payload release, immune activation, and safety.

For intranasal nano-immunotherapy, dosing uncertainty has an additional immunological dimension. Immune agonists, siRNA, CRISPR systems, bacterial vesicles, and checkpoint-modulating payloads may have narrow therapeutic windows. Too little exposure may fail to remodel the tumor immune microenvironment, while excessive exposure may cause nasal inflammation, neuroinflammation, or systemic immune toxicity. Future studies should therefore define not only the maximum tolerated dose but also the minimum immunologically active dose and the optimal biological dose. These parameters may be more relevant than conventional maximum-dose escalation for immune-active nanomedicines.

### 7.3 Human mucosal tolerance and safety considerations

Human mucosal tolerance data for intranasal glioma therapy are currently derived mainly from perillyl alcohol/NEO100 rather than from nanoparticle-based immune formulations (206, 221). In the completed Phase I portion of the NEO100 trial, repeated intranasal administration up to 1152 mg/day was reported to be well tolerated, and the maximum tolerated dose was not reached. No grade 3 or 4 adverse events were observed. Reported grade 1 local or administration-related events included nasal soreness or itching, rhinorrhea, nasal dryness, nasal discomfort, skin irritation around the nose, cough, and headache (206, 208). Earlier Brazilian perillyl alcohol studies also reported good tolerance and high compliance during repeated intranasal administration (222, 223). However, these data should not be overgeneralized to intranasal nanoparticles. Nanocarriers, cationic materials, immune agonists, bacterial vesicles, CRISPR systems, and metal-based platforms may introduce additional mucosal, olfactory, inflammatory, and systemic risks that were not captured by small-molecule perillyl alcohol studies.

Safety is a central translational concern for intranasal nano-immunotherapy. The nasal cavity is not merely a passive entry site; it is an immunologically active mucosal surface with epithelial cells, mucus, cilia, resident immune cells, sensory neurons, and olfactory structures. Repeated exposure to nanoparticles, immune agonists, cationic carriers, bacterial vesicles, or gene-editing systems may cause epithelial irritation, ciliary dysfunction, mucosal inflammation, altered smell, or local barrier disruption (224). Therefore, nasal mucosal safety should be evaluated systematically rather than assumed. Olfactory safety is especially important because the same anatomical route that enables nose-to-brain delivery may expose olfactory neurons and olfactory bulb structures to immune-active materials. Damage to the olfactory epithelium or olfactory bulb could lead to impaired smell, local neuroinflammation, or broader neurological effects. Preclinical studies should include histological evaluation of the nasal mucosa, olfactory epithelium, olfactory bulb, and adjacent brain regions after repeated dosing. Behavioral tests related to olfaction and neurological function should also be considered, particularly for formulations intended for chronic or repeated administration.

Immune agonist-based nanomedicines require careful inflammatory safety assessment. cGAS-agonistic spherical nucleic acids can reprogram the GBM immune microenvironment and promote antitumor immunity, while STING-related sequential-release systems can restore immune activation in GBM, and polyinosinic/polycytidylic lipid nanoparticles can enhance immune-cell infiltration and survival (101, 107). These strategies are mechanistically attractive, but STING and TLR activation may also induce excessive type I interferon signaling, cytokine release, microglial activation, edema, or neurotoxicity if not properly controlled. Therefore, immune activation should be evaluated together with inflammatory toxicity in tumor-bearing and non-tumor-bearing animals. Gene-silencing and genome-editing systems raise additional safety questions. T7 peptide-mediated siRNA nanomicelles delivered intranasally provide a direct example of nucleic acid-based glioma immunotherapy (32). CRISPR-Cas9 nanoparticles targeting GDF15 demonstrate the potential of genome editing to enhance GBM immunotherapy (108). However, CRISPR systems require evaluation of off-target editing, persistence of editing components, immune responses to Cas proteins, unintended effects on normal brain cells, and long-term genomic safety. For intranasal use, it is also important to determine whether editing components accumulate in non-tumor brain regions or peripheral organs. Bacterial outer membrane vesicle-based platforms present another safety challenge. OMV-based co-delivery of doxorubicin and CD47-siRNA can overcome immune resistance and potentiate GBM immunotherapy (109). However, OMVs may contain pathogen-associated molecular patterns that stimulate innate immunity. While this may enhance antitumor responses, excessive activation may cause inflammation, fever-like responses, neuroinflammation, or systemic toxicity. Endotoxin content, vesicle purification, detoxification strategies, and batch-to-batch consistency must therefore be carefully controlled.

Safety evaluation should be organized into local, central, and systemic layers. Local safety should include nasal mucosal histology, epithelial integrity, ciliary function, mucus production, olfactory epithelial injury, and inflammatory-cell infiltration because nasal deposition, mucociliary clearance, and prolonged mucosal exposure are important route-specific determinants of both delivery performance and tolerability (94). CNS safety should cover microglial activation, astrocytosis, neuronal injury, demyelination, edema, seizure-like behavior, neurological dysfunction, and brain cytokine induction, particularly for immune-active or inflammatory nanoplatforms that may access the CNS through olfactory or trigeminal pathways (225). Systemic safety should include body weight, hematology, liver and kidney function, serum cytokines, spleen activation, complement activation, and major-organ histology; complement responses are particularly relevant because nanoparticle composition and surface chemistry can substantially influence inflammatory and systemic toxicity (226). Repeated-dose tolerability should be considered a prerequisite for advancement because the anticipated clinical use of intranasal nano-immunotherapy is likely to involve repeated or cyclic administration. Persistent mucosal injury, olfactory dysfunction, progressive neuroinflammation, neurological deficits, or clinically meaningful systemic immune toxicity should be treated as major no-go signals even when antitumor efficacy is observed. Such a multilayered and repeated-dose safety framework is therefore necessary for establishing translational readiness of intranasal nanomedicine rather than inferring safety from short-term antitumor efficacy alone (205).

### 7.4 Manufacturing and reproducibility

Manufacturing is another major barrier to clinical translation. Many experimental nanoplatforms are complex, multi-component systems that include targeting ligands, nucleic acids, immune agonists, membrane coatings, bacterial vesicles, metals, photosensitizers, or controlled-release structures (227, 228). While these designs are scientifically attractive, each additional component increases the difficulty of reproducible production, quality control, sterilization, storage, and regulatory approval. Therefore, translational potential should be considered during platform design rather than after efficacy has been demonstrated. Particle-size consistency is a key requirement. Nanoparticle size and polydispersity influence nasal deposition, mucus diffusion, epithelial uptake, brain distribution, cellular internalization, and immune activation. Small changes in size or surface charge can alter biodistribution and toxicity. For platforms such as cGAS-agonistic spherical nucleic acids, biomimetic nanoparticles, and biomimetic immune-remodeling nanoparticles, reproducible nanoscale architecture is essential for consistent biological activity (17, 73). Batch-to-batch variation could lead to differences in immune activation, tumor targeting, or safety.

Payload loading and release kinetics also require strict control. Nucleic acid payloads must be protected from degradation while remaining available for intracellular release. Immune agonists must be released at levels sufficient to activate antitumor immunity without causing excessive inflammation. Combination systems, such as engineered extracellular vesicles used for radiation-primed checkpoint inhibition or OMV-based co-delivery of doxorubicin and CD47-siRNA, require accurate control of payload ratio (109, 120). If one payload is released too early or too late, the intended synergy may be lost. Sterility and endotoxin control are especially important for intranasal and immune-active nanomedicines. Intranasal formulations contact mucosal surfaces and may access the central nervous system. Contamination could therefore produce serious inflammatory complications. OMV-based systems require particularly rigorous endotoxin characterization and detoxification. Biomimetic or membrane-coated systems require standardized source materials, purification procedures, and identity testing. Extracellular vesicle-based formulations require control of vesicle heterogeneity, protein/RNA content, and residual impurities.

Long-term storage and formulation stability also influence clinical feasibility. Nanoparticles may aggregate, lose payload, change surface properties, or degrade during storage (229, 230). Nucleic acids may be unstable, proteins may denature, and membrane coatings may change composition during storage, transport, or processing (231). For intranasal use, the final product must also be compatible with nasal spray, droplet, gel, or device-based delivery formats. A formulation that performs well when freshly prepared in a laboratory may not be suitable for clinical use unless it can be stored, transported, and administered reproducibly. Good Manufacturing Practice development should therefore focus on simplification, scalability, and quality-by-design (232, 233). Key release specifications may include particle size, PDI, zeta potential, morphology, sterility, endotoxin level, payload content, encapsulation efficiency, release profile, residual solvent, ligand density, potency assay, and stability. Endotoxin control is particularly important for nanomedicines because nanoparticle properties can interfere with conventional endotoxin assays and complicate quality assessment (234). For immune-active nanomedicines, a functional potency assay is particularly important. For example, a STING-activating platform should show reproducible induction of interferon-related signaling, while a siRNA system should show reproducible target-gene knockdown. Without such assays, it will be difficult to compare batches or define clinically meaningful dosing. For translational advancement, manufacturing reproducibility should therefore be treated as a go/no-go criterion rather than a late-stage regulatory consideration. Candidate formulations should demonstrate predefined acceptance ranges for critical quality attributes across independent production batches, including particle size distribution, PDI, payload content, release behavior, potency, sterility/endotoxin control, and storage stability (235). A platform showing substantial batch-dependent variation in biological potency, biodistribution, or immune activation should not progress solely because one optimized laboratory batch shows strong antitumor efficacy. Scalable manufacturing should ultimately reproduce both physicochemical attributes and biological performance across increasing production scales (236).

### 7.5 Biomarkers and imaging-guided validation

Biomarkers are essential for bridging preclinical nano-immunotherapy and clinical application. In glioma, radiographic response can be difficult to interpret because treatment-related inflammation, edema, necrosis, and pseudoprogression may mimic tumor progression. This challenge becomes even greater for immunotherapy, where immune-cell infiltration and inflammatory changes may temporarily increase lesion size or contrast enhancement. Therefore, intranasal nano-immunotherapy requires biomarkers that can distinguish delivery, immune activation, tumor response, and toxicity. Imaging-guided validation is especially important (237–239). MRI remains the central clinical imaging modality for glioma, but conventional tumor volume measurement may be insufficient. Radiomics, diffusion imaging, perfusion imaging, spectroscopy, and immune-related imaging approaches may provide earlier or more mechanistic information. Nanoparticle-mediated blockade of CXCL12/CXCR4 signaling combined with MRI radiomics provides an important example of how nanotherapy response can be monitored noninvasively (13). This type of strategy is valuable because it links immune-targeted nanotherapy with measurable imaging features that may help predict response before survival differences become evident.

Immune biomarkers should also be incorporated into translational studies. Candidate biomarkers include interferon-stimulated gene signatures, CXCL10 induction, T-cell infiltration markers, macrophage/microglia activation states, PD-L1 expression, CD47 expression, GDF15 expression, CXCL12/CXCR4 activity, cytokine profiles, and antigen-presentation markers (240, 241). For innate immune agonist platforms, STING or TLR pathway activation may serve as pharmacodynamic evidence, provided that pathway activation is demonstrated in the relevant tumor or immune compartment rather than inferred from bulk inflammatory changes (18). For siRNA, CRISPR, innate immune agonist, or checkpoint-modulating systems, target engagement should ideally be demonstrated within the intended cellular compartment rather than only in bulk tumor tissue. For example, tumor-level target knockdown is insufficient if the therapeutic mechanism requires modulation of resident microglia, monocyte-derived macrophages, dendritic cells, or a defined T-cell compartment. Cell sorting, single-cell analysis, spatial profiling, or other cell-resolved pharmacodynamic approaches should therefore be incorporated whenever the proposed mechanism depends on cell-specific activity (242). For checkpoint-modulating systems, changes in T-cell activation and exhaustion states may be more informative than checkpoint expression alone, particularly because progenitor/stem-like and terminally exhausted CD8+ T-cell populations differ substantially in persistence, proliferative potential, and therapeutic responsiveness (167). Spatial assessment may provide additional information on whether these functional immune populations establish sustained interactions with tumor cells rather than merely increasing in overall abundance (201). Circulating and cerebrospinal fluid biomarkers may help overcome the limited availability of serial tumor tissue in GBM patients. Potential sources include plasma cytokines, circulating immune-cell phenotypes, extracellular vesicles, cell-free DNA, tumor-derived RNA, and CSF immune mediators (204, 243). Recent analyses further indicate that tumor-associated extracellular-vesicle populations can be detected in plasma and CSF and may provide complementary molecular information for longitudinal GBM monitoring (244). For intranasal therapy, nasal mucosal biomarkers may also be useful for assessing local inflammation and tolerability. However, peripheral immune changes may not always reflect intracranial immune remodeling, so biomarker interpretation should be linked with imaging and, when possible, tissue analysis. MRI-derived radiogenomic and other advanced imaging biomarkers may complement molecular and immune assays, although prospective validation remains limited (245). A clinically useful biomarker strategy should answer several questions. First, did the nanomedicine reach the brain tumor region? Second, did it activate the intended immune pathway? Third, did it remodel the tumor immune microenvironment rather than only cause nonspecific inflammation? Fourth, did it produce early evidence of tumor control? Fifth, did it cause local nasal, neurological, or systemic toxicity? Answering these questions will require integrated biomarker panels rather than a single endpoint. Imaging, immune profiling, pharmacodynamic assays, and safety biomarkers should therefore be incorporated into preclinical and early-phase clinical trial designs.

### 7.6 Critical limitations and risk-adjusted interpretation of therapeutic potential

The therapeutic potential of intranasal nano-immunotherapy should be interpreted through a risk-adjusted framework rather than by antitumor efficacy alone (145, 246). A central uncertainty is delivery partitioning after intranasal administration. Total brain accumulation does not necessarily indicate therapeutically meaningful tumor delivery. A formulation may accumulate in the nasal mucosa, olfactory bulb, normal brain parenchyma, cerebrospinal fluid-associated spaces, perivascular compartments, blood, liver, spleen, or kidney without reaching sufficient concentrations in infiltrative glioma regions (247, 248). Therefore, future studies should quantify not only total brain exposure, but also the fraction of administered material that reaches tumor tissue relative to normal brain and systemic organs. Key metrics should include nasal retention, olfactory bulb exposure, tumor-to-normal brain ratio, tumor-to-blood ratio, liver and spleen accumulation, kidney clearance, and duration of tumor-associated retention. For advancement, biodistribution should therefore demonstrate reproducible tumor enrichment relative to normal brain rather than merely detectable whole-brain accumulation. The tumor-to-normal brain ratio should be reported quantitatively together with inter-animal variability, systemic exposure, regional brain distribution, and retention over time. A formulation that predominantly accumulates in the olfactory bulb or normal brain without consistent tumor enrichment should not be considered ready for further translational development.

A second limitation is variability in nasal deposition and dosing. Intranasal delivery is strongly influenced by administration volume, droplet size, formulation viscosity, head position, anesthesia, breathing pattern, mucociliary clearance, mucus thickness, nasal inflammation, and device design (52, 54, 218). In rodent studies, intranasal dosing is often performed under controlled anesthesia or restraint, whereas human administration would depend on sprays, nebulizers, nasal masks, or device-assisted deposition (53, 249). Therefore, apparent therapeutic efficacy in rodents may reflect optimized experimental deposition rather than a robust delivery principle that can be directly reproduced in patients. Dose-response studies, deposition mapping, repeated-dose tolerability, and device-compatible formulation testing should be incorporated before claiming translational potential.

A third limitation is species-specific nasal anatomy. Rodents have a proportionally larger olfactory epithelial region than humans, which may overestimate the efficiency of direct nose-to-brain transport in murine models. Differences in turbinate complexity, airflow pattern, nasal cavity size, mucociliary clearance, and olfactory surface area can alter where nanoparticles deposit and how efficiently they reach the brain (214, 215). Therefore, murine nose-to-brain delivery should be considered proof-of-concept rather than direct evidence of human delivery efficiency. Large-animal models, human nasal cast systems, computational fluid dynamics, and clinically relevant delivery devices may help bridge this anatomical gap.

A fourth and particularly important risk is immune activation without meaningful antitumor benefit. Intranasal delivery of STING agonists, TLR agonists, bacterial vesicles, cationic nanoparticles, or gene-editing systems may activate local innate immunity in the nasal mucosa, olfactory structures, normal brain, or perivascular compartments. Such activation could produce cytokine release, microglial activation, astrocytosis, edema, olfactory dysfunction, or neuroinflammation even if tumor delivery is insufficient (246, 250, 251). Therefore, immune pharmacodynamic validation should distinguish productive antitumor immunity from nonspecific inflammation. Evidence of interferon signaling, cytokine induction, or immune-cell infiltration should be interpreted together with tumor localization, antigen presentation, CD8+ T-cell function, phagocytosis, survival benefit, recurrence control, and neurological safety.

These limitations should be explicitly considered when evaluating different nanoplatforms. Highly immunostimulatory systems may provide strong immune activation but carry greater neuroinflammatory risk (246, 252). Biomimetic or extracellular-vesicle-based systems may improve biological interactions but face heterogeneity and manufacturing challenges (73, 253). Gene-silencing or genome-editing platforms may offer target specificity but require evidence of tumor-localized knockdown or editing (46). Stimuli-responsive systems may create mechanistic synergy but often add complexity, device dependence, or lesion-specific constraints (132). Thus, future platform evaluation should integrate delivery efficiency, immune mechanism, efficacy signal, biodistribution uncertainty, inflammatory safety, and translational readiness rather than focusing only on tumor inhibition.

### 7.7 Translational pathway and practical advancement criteria toward first-in-human studies

A realistic translational pathway for intranasal nano-immunotherapy should proceed through several sequential development stages rather than moving directly from murine efficacy to clinical testing (185, 186, 254). Importantly, these stages should not be viewed merely as a descriptive development sequence. Advancement to more resource-intensive translational studies should depend on predefined go/no-go criteria that demonstrate reproducible product quality, route-specific delivery, mechanism-linked immune pharmacodynamics, therapeutic efficacy, and repeated-dose safety. The first step is formulation standardization. Candidate nanoplatforms should be defined by critical quality attributes, including particle size distribution, polydispersity index, surface charge, morphology, payload loading, encapsulation efficiency, release kinetics, sterility, endotoxin level, residual solvent or reagent content, storage stability, and batch-to-batch reproducibility (187, 255). For biomimetic, extracellular-vesicle, bacterial-vesicle, or nucleic-acid-based systems, additional release criteria should include membrane-source characterization, vesicle purity, nucleic-acid integrity, immune agonist potency, endotoxin control, and biological activity after storage.

The second step is GMP-compatible manufacturing and chemistry, manufacturing, and controls development. Intranasal nanoformulations intended for human use should be scalable, reproducible, sterile or appropriately controlled for microbial burden, and compatible with a defined nasal delivery device (255–257). Regulatory expectations for nanomaterial-containing drug products emphasize detailed characterization, manufacturing control, and product-specific assessment, while nasal spray or inhalation products require careful CMC documentation of formulation, container closure, device performance, and delivered dose uniformity (258). Therefore, early development should avoid overly complex platforms unless their added biological function clearly improves delivery, immune pharmacodynamics, or safety.

The third step is route-specific toxicology. General toxicology should be supplemented with intranasal-route-specific endpoints, including nasal mucosal histology, epithelial integrity, ciliary function, olfactory epithelial injury, olfactory bulb exposure, neuroinflammation, behavioral safety, systemic cytokine release, and major-organ exposure (259). For immune agonists, bacterial vesicles, cationic carriers, and gene-editing systems, toxicology should also assess excessive innate immune activation, complement activation, off-target immune stimulation, and persistence of the payload or carrier (246, 260, 261). Nonclinical safety packages should be aligned with general first-in-human principles and oncology-specific nonclinical guidance when applicable.

The fourth step is device and dosing standardization. Intranasal translation cannot rely only on pipette-based dosing in anesthetized rodents. Human development requires a defined delivery device, droplet or aerosol size distribution, spray plume geometry, emitted dose, delivered dose, nasal deposition pattern, dosing frequency, head position, and compatibility between formulation viscosity and device performance (53, 217, 262). Repeated-dose tolerability should be evaluated because glioma therapy would likely require chronic or cyclic administration rather than single dosing (145, 206).

The fifth step is pharmacokinetic and pharmacodynamic bridging. Before first-in-human testing, studies should quantify the fraction of administered material retained in the nasal cavity, delivered to normal brain, accumulated in tumor tissue, absorbed systemically, or cleared through liver, spleen, and kidney (247, 263). Pharmacodynamic biomarkers should then determine whether the formulation reaches the intended immune compartment and activates the intended mechanism. Candidate biomarkers may include target-gene knockdown or editing, STING/TLR pathway activation, interferon-stimulated gene signatures, CXCL10 induction, PD-L1 or CD47 modulation, macrophage/microglia phagocytosis, dendritic-cell activation, CD8+ T-cell infiltration, cytokine profiles, MRI-based response markers, and nasal mucosal inflammation markers.

The final step is first-in-human trial design. A realistic first-in-human study should start with a simple and well-characterized formulation, a justified starting dose, careful dose escalation, predefined stopping rules, nasal and neurological safety monitoring, pharmacokinetic sampling when feasible, imaging-based assessment of brain/tumor exposure, and pharmacodynamic biomarker collection (185, 186). Early trials should prioritize safety, mucosal tolerability, device feasibility, systemic exposure, and immune pharmacodynamic evidence before making efficacy claims. Accordingly, progression toward formal translational development should be based on predefined advancement criteria rather than antitumor activity alone. A candidate intranasal nano-immunotherapy should demonstrate (1): a reproducible formulation with predefined critical quality attributes and acceptable batch-to-batch consistency (2); quantitative and reproducible tumor enrichment relative to normal brain, together with characterization of systemic exposure (3); cell-specific uptake and molecular target engagement in the intended tumor or immune-cell compartment (4); a reproducible survival benefit in immunocompetent orthotopic glioma models rather than tumor-volume reduction alone (5); spatially resolved immune pharmacodynamics demonstrating functionally relevant tumor–immune interactions (6); acceptable repeated-dose nasal, olfactory, neurological, and systemic safety; and (7) a scalable and reproducible manufacturing process compatible with GMP/CMC development and a clinically relevant nasal delivery device. These criteria should be considered collectively. Strong performance in one domain should not compensate for a critical deficiency in another. For example, high total brain exposure should not justify further development if tumor-to-normal brain selectivity is poor; tumor accumulation should not be considered sufficient if the intended cellular target is not engaged; and immune activation should not support advancement if it is accompanied by unacceptable neuroinflammation or if it fails to produce durable survival benefit. Likewise, a biologically sophisticated nanoplatform should not progress solely on the basis of efficacy if its composition, potency, or manufacturing characteristics cannot be reproduced across batches. We therefore propose a practical go/no-go framework in which advancement requires convergence of product quality, quantitative delivery, target engagement, immune pharmacodynamics, efficacy, safety, and manufacturability. Only candidates that establish this integrated evidence package should be prioritized for large-animal bridging studies, GMP-compatible process development, regulatory toxicology, and ultimately first-in-human evaluation.

## 8 Future directions and hypothesis-generating concepts

Intranasal nano-immunotherapy remains at an early preclinical stage. Therefore, the concepts discussed in this section should be interpreted as future directions and hypothesis-generating frameworks rather than evidence-based clinical recommendations. In particular, immune-niche modulation, biomarker-guided payload selection, and personalized intranasal nano-immunotherapy are not currently established clinical strategies. Their feasibility will require direct validation of nasal delivery, tumor-selective accumulation, immune-cell-specific pharmacodynamics, safety, and clinically usable biomarkers.

### 8.1 Future testing of immune-niche modulation after nose-to-brain delivery

The next conceptual step is to test whether nose-to-brain delivery can be coupled with spatially defined immune-niche modulation. Earlier intranasal nanomedicine studies have focused mainly on whether drugs can reach the brain or reduce tumor growth. This remains important, but it is no longer sufficient for glioma immunotherapy (230, 264). The key question is whether an intranasal nanoplatform can reproducibly reach glioma-associated compartments and induce beneficial immune changes without causing nonspecific neuroinflammation. GBM contains multiple spatially organized immune niches. TAM-rich niches are dominated by macrophages, microglia, and suppressive myeloid programs. T-cell-excluded niches contain immune barriers that prevent cytotoxic lymphocytes from reaching tumor cells. Hypoxic niches promote metabolic stress, angiogenesis, myeloid suppression, and resistance to therapy. Invasive margins contain infiltrating glioma cells protected by relatively intact BBB and specialized stromal interactions. Perivascular niches support tumor stem-like cells, immune-cell trafficking, and therapeutic resistance. Future intranasal nano-immunotherapy candidates could be designed according to the glioma-associated compartments they are intended to modulate, but this approach requires spatial biodistribution and immune pharmacodynamic validation (45, 265).

Different payloads may be suited to different immune niches. STING or TLR agonists may be useful for activating innate immune sensing and attracting T cells into immune-cold regions. CXCL12/CXCR4 inhibitors may help overcome immune exclusion and suppressive chemokine gradients. CD47-siRNA may be particularly relevant in myeloid-rich niches where macrophage or microglia-mediated phagocytosis can be activated. PD-L1 siRNA or anti-PD-1-enhancing strategies may be most useful when T cells are present but functionally inhibited. ROS/NO-scavenging nanoparticles may help preserve T-cell function in hostile oxidative niches. Thus, future platforms should be designed around immune geography rather than payload availability alone. Spatial technologies will be essential for this transition. Single-cell RNA sequencing, spatial transcriptomics, multiplex immunofluorescence, imaging mass cytometry, and spatial metabolomics can identify which immune niches are present and how they respond to treatment. These tools can help determine whether intranasal nanotherapy reaches the tumor core, invasive margin, perivascular compartment, or normal brain tissue. They can also reveal whether immune activation occurs in productive antitumor regions or in potentially toxic off-target compartments. Therefore, spatial validation should become a standard component of future intranasal nano-immunotherapy studies.

### 8.2 Biomarker-guided design of future intranasal nano-immunotherapy

Biomarker-guided design may be necessary in future studies because gliomas are biologically and immunologically heterogeneous. Tumor genotype, epigenetic state, immune composition, prior treatment exposure, steroid use, and anatomical location may all influence response to intranasal nano-immunotherapy (266, 267). A single formulation is unlikely to work equally well across all glioma subtypes. Personalized nanovaccine concepts may further extend this biomarker-guided strategy by matching tumor- or patient-specific antigens with biomimetic delivery systems and appropriate immune adjuvants (114). In glioma, however, such personalization should be regarded as a future design principle rather than an established intranasal strategy, because route-specific brain delivery, antigen-specific immune pharmacodynamics, manufacturing reproducibility, and safety remain to be demonstrated. In future clinical development, candidate selection may need to be guided by tumor molecular features and immune microenvironment profiles, provided that the delivery platform has first demonstrated safety, brain exposure, and immune pharmacodynamic activity. Classical glioma biomarkers may help stratify patients. IDH-mutant and IDH-wildtype gliomas differ in metabolism, immune infiltration, myeloid composition, and inflammatory signaling. MGMT promoter methylation influences temozolomide sensitivity and may affect the rational use of chemo-immunotherapy combinations. Tumors with high CXCL12/CXCR4 activity may be more suitable for chemokine-axis-targeted nanotherapy. Tumors with high PD-L1 expression or T-cell infiltration may be better candidates for checkpoint-modulating payloads. Tumors with high CD47 expression or macrophage-rich microenvironments may be suited to phagocytosis checkpoint strategies. Tumors with GDF15-associated immune suppression may benefit from gene-editing or gene-silencing platforms targeting this pathway.

Innate immune pathway status should also guide treatment selection. STING pathway competence, interferon signaling capacity, TLR expression, dendritic-cell abundance, and antigen-presentation markers may determine whether innate immune agonists will work. If a tumor has suppressed STING pathway activity, epigenetic reprogramming strategies such as sequential-release STING-restoring systems may be needed before immune agonist delivery (101). If TLR3 signaling is intact, polyinosinic/polycytidylic lipid nanoparticles may provide a rational strategy for immune activation (107). If innate immune activation is already high but T cells remain excluded, chemokine-axis blockade or myeloid reprogramming may be more appropriate. Personalized intranasal nano-immunotherapy will also require adaptive treatment timing. Newly diagnosed tumors, post-radiation tumors, recurrent tumors, and steroid-treated tumors may have different immune states. Radiation may prime tumors for immune checkpoint modulation and nanotherapy, as suggested by engineered extracellular vesicles in radiation-primed GBM and irradiated GBM-tropic nanoplatforms (120, 131). Recurrent tumors may show stronger immune suppression, therapy-induced myeloid remodeling, and altered BBB/BTB properties. Therefore, timing and sequencing may be as important as payload choice. Ultimately, a future biomarker-guided development strategy could integrate molecular pathology, imaging, immune profiling, and delivery feasibility, but this remains a proposed framework rather than a clinically validated workflow. A future clinical workflow could involve defining tumor genotype, measuring immune niche features, identifying dominant suppressive pathways, selecting a matching intranasal nanoplatform, and monitoring response with MRI radiomics and immune biomarkers. This would move the field from empirical nanomedicine toward mechanism-guided precision immunotherapy.

### 8.3 Combination strategies will dominate

Combination therapy is likely to dominate the future of intranasal nano-immunotherapy because GBM resistance is multi-layered. Delivery barriers, tumor-cell heterogeneity, immune exclusion, myeloid suppression, checkpoint activation, hypoxia, metabolic stress, and poor antigen presentation cannot be solved by a single intervention (268). Nanocarriers provide an opportunity to coordinate multiple therapeutic functions within one platform or treatment sequence. Intranasal nanotherapy combined with radiotherapy is one of the most rational directions because radiotherapy is already a standard component of GBM treatment and can induce DNA damage, antigen release, inflammatory signaling, and vascular remodeling (269). Engineered extracellular vesicles have been used in radiation-primed GBM to enhance localized immunomodulation, while an engineered nanoplatform with tropism toward irradiated GBM augmented radioimmunotherapy efficacy (131, 181). These studies suggest that radiotherapy can create a therapeutically favorable context for immune-active nanomedicine. Future intranasal platforms could be administered before, during, or after radiotherapy to enhance antigen presentation, activate innate immunity, or overcome checkpoint-mediated resistance. However, the optimal sequencing and route-specific pharmacodynamics of such combinations require direct intranasal validation.

Intranasal nanotherapy combined with PD-1/PD-L1 blockade is another important direction. T-cell shielding nanoparticles that scavenge nitric oxide/reactive oxygen species can potentiate anti-PD-1 therapy in GBM (133). This suggests that checkpoint blockade may require microenvironmental support to protect T cells from oxidative and metabolic stress. Intranasal platforms could deliver siPD-L1, STING agonists, TLR agonists, or T-cell-protective agents locally, while systemic or local checkpoint blockade sustains T-cell function. Innate immune activation combined with myeloid reprogramming may be especially powerful. STING/TLR agonists can initiate inflammatory priming, but the resulting immune response may still be suppressed by TAMs, MDSCs, or phagocytosis checkpoints. Combining STING or TLR agonists with TAM reprogramming, CXCL12/CXCR4 blockade, CD47 silencing, or metabolic modulation could generate stronger antitumor immunity. Sequential-release STING-related systems, CXCL12/CXCR4-targeted approaches, and OMV/CD47-siRNA platforms provide examples of mechanisms that could be integrated into future designs (13, 101, 109).

Gene-silencing or genome-editing payloads may also be paired with immunogenic cell death induction. CRISPR-Cas9 nanoparticles targeting GDF15 can enhance immunotherapy efficacy in GBM, while OMV-based doxorubicin and CD47-siRNA co-delivery links tumor-cell killing with phagocytosis checkpoint blockade (108, 109). This combination logic is important because immune suppression should be removed at the same time that tumor antigens are released. Without antigen release, checkpoint modulation may be insufficient; without immune suppression reversal, antigen release may fail to generate productive immunity. Physical enhancement strategies may further improve intranasal nanotherapy. Koh et al. showed that intranasal temozolomide-conjugated nanoparticles combined with transcranial magnetic stimulation improved survival in GBM xenograft models (26). Other physical strategies, such as focused ultrasound, magnetic guidance, photodynamic activation, or radiation-induced targeting, may improve brain distribution, tumor penetration, or local immune activation. Photodynamic immunotherapy using a covalent organic framework nanoplatform combined with immune checkpoint blockade also supports the potential of multimodal immune activation (140). The future of intranasal nano-immunotherapy will therefore likely involve rational sequencing rather than simple drug combination. A potential strategy may involve radiation or chemotherapy to release tumor antigens, intranasal STING/TLR agonists to activate innate immunity, siRNA or CRISPR payloads to silence immune-evasion pathways, myeloid-targeting agents to reverse suppression, and checkpoint blockade to sustain T-cell killing. The challenge is to design combinations that are mechanistically synergistic, clinically feasible, and safe for repeated administration.

In conclusion, intranasal nano-immunotherapy represents a promising but still emerging strategy for glioma. Its value lies in the integration of BBB bypass, brain tumor targeting, immune payload delivery, and microenvironmental reprogramming. Future progress will depend on rigorous formulation optimization, spatial immune validation, biomarker-guided patient selection, and rational combination therapy. If these challenges can be addressed, intranasal nano-immunotherapy may become a meaningful bridge between preclinical glioma immunology and clinically translatable brain-directed immunotherapy.

## 9 Conclusion

Intranasal nano-immunotherapy represents a promising strategy for glioma treatment because it integrates three therapeutic concepts that are often addressed separately: bypassing the blood–brain barrier, improving brain tumor-targeted delivery, and reprogramming the immunosuppressive glioma microenvironment. Gliomas, especially GBM, remain difficult to treat not only because of aggressive tumor biology, but also because therapeutic agents often fail to reach invasive intracranial tumor niches and because the local immune ecosystem is dominated by myeloid suppression, T-cell dysfunction, poor antigen presentation, and checkpoint-mediated immune escape. Intranasal nanomedicine may provide a noninvasive route for nose-to-brain delivery, while nanocarriers are designed to offer payload protection, controlled release, tumor targeting, and immune-modulating capacity. Recent preclinical studies have shown that intranasal nanoplatforms can improve brain delivery and prolong survival in glioma models, while nano-immunotherapeutic systems can activate innate immune sensing, silence immune-evasion genes, block chemokine-driven suppression, enhance T-cell function, induce immunogenic cell death, and improve the efficacy of checkpoint blockade. However, the field remains early. Only a limited number of studies directly combine intranasal administration, nanotechnology, glioma targeting, and immune microenvironment reprogramming. Therefore, current evidence should be interpreted as a layered framework rather than as a fully mature therapeutic paradigm. Future progress will require rigorous preclinical validation. Studies should demonstrate not only tumor inhibition, but also nose-to-brain biodistribution, tumor-selective accumulation, immune pharmacodynamics, orthotopic efficacy, survival benefit, recurrence control, and safety in the nasal mucosa, olfactory system, brain, and peripheral organs. Immunocompetent orthotopic models, spatial immune profiling, single-cell analysis, imaging-guided monitoring, and clinically relevant toxicity assessment will be essential for determining whether intranasal nano-immunotherapy can be translated into patient care. Overall, intranasal nano-immunotherapy should not be viewed merely as an alternative delivery route. It is better understood as a brain-directed immune-engineering strategy that may transform inaccessible and immune-cold glioma niches into therapeutically responsive immune environments. If supported by rigorous pharmacokinetic, immune pharmacodynamic, safety, and early-phase clinical evidence, this approach may eventually help bridge the gap between preclinical glioma immunotherapy and clinically testable brain-directed treatment strategies.
