# Supplementary material for: Intranasal nano-immunotherapy for glioma: bypassing the blood–brain barrier, reprogramming the immune microenvironment, and bridging preclinical validation
Source: Front Immunol. 2026 Oct 20;17:1950362. doi: 10.3389/fimmu.2026.1950362 (PMC13728787; doi:10.3389/fimmu.2026.1950362)
Supplement: Supplementary file 1 [file Table1.docx]

**Table S1. Route-specific evidence for intranasal and brain-targeted nanoplatforms in glioma**

| **Nanoplatform** | **Payload** | **Administration route** | **Evidence level** | **Targeting/delivery rationale** | **Glioma model** | **Key outcome** | **PMID** |
| --- | --- | --- | --- | --- | --- | --- | --- |
| T7 peptide-guided self-assembled nanomicelle | siRNA | Intranasal | Level I | T7-mediated brain-tumor targeting; protects siRNA and supports nose-to-brain delivery | Glioma immunotherapy model | Improved tumor-directed siRNA delivery and antitumor immune activity | [1] |
| Targeted lipid-based nanocarrier | Chemotherapeutic agents | Intranasal | Level II | Enhances intracranial drug access while limiting systemic exposure | Intracranial GBM mouse model | Suppressed GBM growth and prolonged survival | [2] |
| Ferritin-based nanovectors | Anticancer drug cargo | Intranasal (nose-to-brain) | Level II | Endogenous carrier-inspired design supports glioma-selective brain delivery | Glioma-bearing preclinical models | Reduced tumor growth and improved survival | [3] |
| Biomimetic nanoparticles | GBM-targeted therapeutic cargo | Intranasal (nose-to-brain) | Level II | Supports mucosal interaction, controlled release, and GBM-targeted delivery | In vitro and preclinical GBM validation | Supported GBM-targeted nose-to-brain delivery | [4] |
| Temozolomide-conjugated nanoparticles | Temozolomide | Intranasal | Level II | TMS-assisted enhancement of nanoparticle transport or therapeutic performance | GBM xenograft models | Improved survival | [5] |
| cGAS-agonistic spherical nucleic acids | cGAS agonist / immunostimulatory nucleic acid | Non-intranasal | Level III | Enhances innate immune activation; supports future intranasal adaptation | GBM immune microenvironment models | Reprogrammed the GBM immune microenvironment and promoted antitumor immunity | [6] |

Evidence levels: Level I, direct intranasal glioma nano-immunotherapy evidence; Level II, intranasal glioma nanodelivery without demonstrated immune reprogramming; Level III, non-intranasal glioma nano-immunotherapy supporting future intranasal adaptation. Abbreviations: cGAS, cyclic GMP-AMP synthase; GBM, glioblastoma; siRNA, small interfering RNA; TMS, transcranial magnetic stimulation.

1.	Tang L, Zhang R, Wang Y, Zhang X, Yang Y, Zhao B, Yang L: **A simple self-assembly nanomicelle based on brain tumor-targeting peptide-mediated siRNA delivery for glioma immunotherapy via intranasal administration**. *Acta biomaterialia* 2023, **155**:521-537.

2.	Sandbhor P, Goda J, Mohanty B, Gera P, Yadav S, Chekuri G, Chaudhari P, Dutt S, Banerjee R: **Targeted nano-delivery of chemotherapy via intranasal route suppresses in vivo glioblastoma growth and prolongs survival in the intracranial mouse model**. *Drug delivery and translational research* 2023, **13**(2):608-626.

3.	Marrocco F, Falvo E, Mosca L, Tisci G, Arcovito A, Reccagni A, Limatola C, Bernardini R, Ceci P, D'Alessandro G *et al*: **Nose-to-brain selective drug delivery to glioma via ferritin-based nanovectors reduces tumor growth and improves survival rate**. *Cell death & disease* 2024, **15**(4):262.

4.	Ferreira NN, Leite CM, Moreno NS, Miranda RR, Pincela Lins PM, Rodero CF, de Oliveira Junior E, Lima EM, Reis RM, Zucolotto V: **Nose-to-Brain Delivery of Biomimetic Nanoparticles for Glioblastoma Targeted Therapy**. *ACS applied materials & interfaces* 2025, **17**(1):484-499.

5.	Koh M, Lee JE, Ye E, Lee S, Kim HL, Song HE, Yoo HJ, Kim WJ, Park SM, Yang SH: **Intranasal administration of temozolomide conjugated nanoparticles with transcranial magnetic stimulation improves the survival in glioblastoma xenograft models**. *Drug delivery and translational research* 2026, **16**(2):509-521.

6.	Mahajan AS, Dussold C, Kim S, Jarvis R, Hurley LA, Tommasini-Ghelfi S, Park J, Forsyth CM, Zhang B, Miska J *et al*: **cGAS-agonistic spherical nucleic acids reprogram the glioblastoma immune microenvironment and promote antitumor immunity**. *Proceedings of the National Academy of Sciences of the United States of America* 2025, **122**(45):e2409557122.
