# Supplementary material for: Intranasal nano-immunotherapy for glioma: bypassing the blood–brain barrier, reprogramming the immune microenvironment, and bridging preclinical validation
Source: Front Immunol. 2026 Oct 20;17:1950362. doi: 10.3389/fimmu.2026.1950362 (PMC13728787; doi:10.3389/fimmu.2026.1950362)
Supplement: Supplementary file 2 [file Table2.docx]

**Table S2. Route-specific evidence, immune mechanisms, and translational gaps of nano-immunotherapeutic strategies for glioma**

| **Nanoplatform/system** | **Immune payload/target** | **Administration route** | **Evidence level** | **Principal target cell/compartment** | **Main immune mechanism** | **Key outcome** | **Major limitation / intranasal translational gap** | **PMID** |
| --- | --- | --- | --- | --- | --- | --- | --- | --- |
| cGAS-agonistic spherical nucleic acids | cGAS/STING activation | Intranasal or intratumoral | Level I | Tumor-associated innate immune cells; T-cell compartment | Activates cGAS-STING, type I IFN signaling, myeloid reprogramming, and T-cell recruitment | Inhibited tumor growth and promoted long-term survival and antitumor immunity | Direct intranasal evidence, but route-specific dosing, spatial PD, and repeated-dose nasal/neurological safety remain to be established | [1] |
| Sequential-release HydroLipo system | Decitabine + STING agonist | Local/in situ | Level III | GBM cells and tumor immune compartment | Restores tumor-cell STING competence by epigenetic reprogramming before immune activation | Reduced tumor growth and postsurgical recurrence while enhancing antitumor immunity | No intranasal validation; local hydrogel delivery and multicomponent release increase route and manufacturing complexity | [2] |
| Poly(I:C) lipid nanoparticles | poly(I:C) ± doxorubicin | Intratumoral/intracranial | Level III | Dendritic cells, macrophages, and T cells | TLR3-associated innate activation with immune-cell infiltration and activation | Enhanced immune-cell infiltration/activation and improved survival in a GBM mouse model | Requires invasive local administration; intranasal delivery, regional CNS exposure, and nasal safety remain untested | [3] |
| T7 peptide-guided nanomicelle | siRNA | Intranasal | Level I | Glioma cells / targeted tumor compartment | Enables nose-to-brain siRNA delivery and target-gene silencing in intracranial glioma | Supported intranasal siRNA-based glioma immunotherapy | Needs broader validation of cell-specific target engagement, immune-state PD, human-relevant dosing, and repeated-dose safety | [4] |
| CXCR4-targeted nitric oxide nanoparticles | PD-L1 siRNA + NO donor | Systemic (non-intranasal) | Level III | CXCR4+ GBM cells; downstream CD8+ T-cell compartment | Silences PD-L1 while NO improves BBB/tumor-vessel permeability and delivery | Increased cytotoxic T-cell infiltration/activation and suppressed GBM progression | No intranasal validation; efficacy depends on cytosolic siRNA delivery and cell-specific PD-L1 knockdown | [5] |
| TME-responsive CRISPR-Cas9 nanoparticles | GDF15 gene editing | Systemic (non-intranasal) | Level III | GBM cells; downstream immune compartment | Edits GDF15 to relieve tumor-driven immunosuppression and enhance checkpoint therapy | Reversed immune suppression and enhanced ICB efficacy | No intranasal validation; off-target editing, persistence, normal-brain exposure, and long-term genomic safety require evaluation | [6] |
| Engineered bacterial outer-membrane vesicles | Doxorubicin + CD47-siRNA | Systemic (non-intranasal) | Level III | GBM cells; macrophages and microglia | Combines tumor stress with CD47 silencing, phagocytic activation, and intrinsic immune adjuvancy | Overcame immune resistance and potentiated GBM immunotherapy | No intranasal validation; OMV immunogenicity/endotoxin control, inflammatory safety, and batch reproducibility are key barriers | [7] |
| Biomimetic nanoparticles | TIME remodeling / PD-L1-related modulation | Systemic (non-intranasal) | Level III | Tumor cells, myeloid cells, Tregs, and CD8+ T cells | Coordinates ICD, metabolic/checkpoint modulation, and broad TIME remodeling | Remodeled the immunosuppressive GBM microenvironment and improved immune control | No intranasal validation; multicomponent complexity, stimulus dependence, source standardization, and manufacturing reproducibility limit translation | [8] |
| Engineered extracellular vesicles | PD-L1 siRNA / checkpoint modulation | Systemic (non-intranasal) | Level III | GBM cells; tumor-associated myeloid and T-cell compartments | Radiation enhances EV tumor accumulation; PD-L1 silencing restores checkpoint responsiveness | Improved immune checkpoint therapy and prolonged survival in radiation-primed GBM | No intranasal validation; EV heterogeneity, cargo-loading consistency, scalable manufacturing, and radiation dependence remain concerns | [9] |
| Synthetic protein nanoparticles | CXCR4/CXCL12 inhibitor (AMD3100) | Systemic | Level III | CXCR4+ tumor cells and monocytic MDSCs | Blocks CXCL12/CXCR4-driven tumor growth, suppressive myeloid recruitment, and immune exclusion | Reduced M-MDSC infiltration, induced ICD, and sensitized tumors to radiotherapy | No intranasal validation; systemic BBB delivery and cell-specific target engagement must be reproduced after nasal administration | [10] |
| Nanoparticle-mediated CXCL12/CXCR4 blockade | AMD3100 + PD-L1 inhibitor BMS-1 | Systemic (non-intranasal) | Level III | Tumor-associated myeloid cells, Tregs, CD8+ T cells, and tumor cells | Combines chemokine-axis blockade with checkpoint inhibition to relieve immune exclusion/suppression | Enhanced GBM immunotherapy and enabled MRI-radiomics monitoring of early response | No intranasal validation; dual-payload complexity and spatial/cell-specific PD require route-specific confirmation | [11] |
| Bacteria-mediated metformin-loaded peptide hydrogel | Metformin + bacterial immune cues | Local/intracranial | Level III | GBM cells, macrophages, and CD8+ T cells | Coordinates metabolic modulation, bacterial immune stimulation, and sustained local immune remodeling | Reprogrammed the GBM immune microenvironment and produced strong intracranial antitumor effects | Invasive/local strategy; bacterial biosafety, product consistency, and compatibility with intranasal delivery remain unresolved | [12] |
| Copper-coordination brain-targeting nanoassembly | Cuproptosis/PDT + thymopentin | Systemic (non-intranasal) | Level III | GBM cells, dendritic cells, and T cells | Induces mitochondrial stress/cuproptosis, activates cGAS-STING, decreases PD-L1, and amplifies adaptive immunity | Improved GBM immunotherapy through TIME reprogramming | No intranasal validation; light dependence, metal-related safety, regional brain exposure, and manufacturing complexity require evaluation | [13] |
| NO/ROS-scavenging nanoparticles | NO/ROS scavenging + anti-PD-1 | Systemic (non-intranasal) | Level III | CD8+ T cells and myeloid-derived oxidative/nitrosative niche | Protects T cells from suppressive NO/ROS and improves checkpoint responsiveness | Increased T-cell infiltration/activation and potentiated anti-PD-1 therapy | No intranasal validation; durable T-cell-state restoration, regional exposure, and route-specific safety remain unproven | [14] |
| Irradiated GBM-tropic biomimetic nanoplatform | PD-L1 antibody + radiosensitizing core | Systemic (non-intranasal) | Level III | Irradiated GBM niche and checkpoint-responsive T-cell compartment | Uses radiation-induced CCL2 tropism for targeted delivery and synergistic radioimmunotherapy | Augmented efficacy and specificity of GBM radioimmunotherapy with reduced irAEs | No intranasal validation; requires radiation priming and complex biomimetic manufacturing | [15] |
| β-Ketoenamine covalent-organic-framework nanoplatform | TMZ + OSMI-4; PDT/checkpoint modulation | Systemic (non-intranasal) | Level III | GBM cells and checkpoint-responsive T-cell compartment | Combines photodynamic tumor damage with O-GlcNAc/PD-L1 regulation and checkpoint-sensitive immunity | Inhibited GBM progression through multimodal photodynamic immunotherapy | No intranasal validation; light penetration, complex formulation, route-specific biodistribution, and scalable manufacturing remain barriers | [16] |

**Evidence levels:** Level I, direct intranasal glioma nano-immunotherapy evidence; Level II, intranasal glioma nanodelivery without demonstrated immune reprogramming; Level III, non-intranasal glioma nano-immunotherapy supporting future intranasal adaptation.

**Abbreviations:** BBB, blood-brain barrier; cGAS, cyclic GMP-AMP synthase; COF, covalent organic framework; CRISPR-Cas9, clustered regularly interspaced short palindromic repeats/CRISPR-associated protein 9; EV, extracellular vesicle; GBM, glioblastoma; GDF15, growth differentiation factor 15; ICB, immune checkpoint blockade; IFN, interferon; LNP, lipid nanoparticle; MDSC, myeloid-derived suppressor cell; MRI, magnetic resonance imaging; NO, nitric oxide; OMV, outer-membrane vesicle; PD, pharmacodynamics; PD-1, programmed cell death protein 1; PD-L1, programmed death-ligand 1; PDT, photodynamic therapy; ROS, reactive oxygen species; siRNA, small interfering RNA; STING, stimulator of interferon genes; TIME, tumor immune microenvironment; TLR3, Toll-like receptor 3; TMZ, temozolomide.

1.	Mahajan AS, Dussold C, Kim S, Jarvis R, Hurley LA, Tommasini-Ghelfi S, Park J, Forsyth CM, Zhang B, Miska J *et al*: **cGAS-agonistic spherical nucleic acids reprogram the glioblastoma immune microenvironment and promote antitumor immunity**. *Proceedings of the National Academy of Sciences of the United States of America* 2025, **122**(45):e2409557122.

2.	Yu H, Liu W, Ding K, Wu J, Wang C, Wang S, Wu L, Tang Q, Yin X, Jiang K *et al*: **Sequential Release HydroLipo System for STING Gene Epigenetic Reprogramming and Immune Activation in Glioblastoma**. *Advanced science (Weinheim, Baden-Wurttemberg, Germany)* 2025, **12**(5):e2408323.

3.	Brüßeler MMT, Zam A, Moreno-Zafra VM, Rouatbi N, Hassuneh OWM, Marrocu A, Liam-Or R, Abdel-Bar HM, Walters AA, Al-Jamal KT: **Polyinosinic/Polycytidylic Lipid Nanoparticles Enhance Immune Cell Infiltration and Improve Survival in the Glioblastoma Mouse Model**. *Molecular pharmaceutics* 2024, **21**(12):6339-6352.

4.	Tang L, Zhang R, Wang Y, Zhang X, Yang Y, Zhao B, Yang L: **A simple self-assembly nanomicelle based on brain tumor-targeting peptide-mediated siRNA delivery for glioma immunotherapy via intranasal administration**. *Acta biomaterialia* 2023, **155**:521-537.

5.	Hsieh HT, Huang HC, Chung CW, Chiang CC, Hsia T, Wu HF, Huang RL, Chiang CS, Wang J, Lu TT *et al*: **CXCR4-targeted nitric oxide nanoparticles deliver PD-L1 siRNA for immunotherapy against glioblastoma**. *Journal of controlled release : official journal of the Controlled Release Society* 2022, **352**:920-930.

6.	Zou C, Liu X, Wang W, He L, Yin A, Cao Z, Zhu M, Wu Y, Liu X, Ma J *et al*: **Targeting GDF15 to enhance immunotherapy efficacy in glioblastoma through tumor microenvironment-responsive CRISPR-Cas9 nanoparticles**. *Journal of nanobiotechnology* 2025, **23**(1):126.

7.	You H, Zhang S, Zhang Y, Chen Q, Wu Y, Zhou Z, Zhao Z, Su B, Li X, Guo Y *et al*: **Engineered Bacterial Outer Membrane Vesicles-Based Doxorubicin and CD47-siRNA Co-Delivery Nanoplatform Overcomes Immune Resistance to Potentiate the Immunotherapy of Glioblastoma**. *Advanced materials (Deerfield Beach, Fla)* 2025, **37**(15):e2418053.

8.	Wang T, Zhang H, Qiu W, Han Y, Liu H, Li Z: **Biomimetic nanoparticles directly remodel immunosuppressive microenvironment for boosting glioblastoma immunotherapy**. *Bioactive materials* 2022, **16**:418-432.

9.	Tian T, Liang R, Erel-Akbaba G, Saad L, Obeid PJ, Gao J, Chiocca EA, Weissleder R, Tannous BA: **Immune Checkpoint Inhibition in GBM Primed with Radiation by Engineered Extracellular Vesicles**. *ACS nano* 2022, **16**(2):1940-1953.

10.	Alghamri MS, Banerjee K, Mujeeb AA, Mauser A, Taher A, Thalla R, McClellan BL, Varela ML, Stamatovic SM, Martinez-Revollar G *et al*: **Systemic Delivery of an Adjuvant CXCR4-CXCL12 Signaling Inhibitor Encapsulated in Synthetic Protein Nanoparticles for Glioma Immunotherapy**. *ACS nano* 2022, **16**(6):8729-8750.

11.	Wei R, Li J, Lin W, Pang X, Yang H, Lai S, Wei X, Jiang X, Yuan Y, Yang R: **Nanoparticle-mediated blockade of CXCL12/CXCR4 signaling enhances glioblastoma immunotherapy: Monitoring early responses with MRI radiomics**. *Acta biomaterialia* 2024, **177**:414-430.

12.	Zhu L, Liu J, Qiu M, Chen J, Liang Q, Peng G, Zou Z: **Bacteria-mediated metformin-loaded peptide hydrogel reprograms the tumor immune microenvironment in glioblastoma**. *Biomaterials* 2022, **288**:121711.

13.	Chen Y, Tian H, Zhang X, Nice EC, Huang C, Zhang H, Zheng S: **Copper-coordination driven brain-targeting nanoassembly for efficient glioblastoma multiforme immunotherapy by cuproptosis-mediated tumor immune microenvironment reprogramming**. *Journal of nanobiotechnology* 2024, **22**(1):801.

14.	Lee J, Kang Y, Lee H, Saravanakumar G, Park SA, Ahn S, Kim WJ: **Amplifying glioblastoma immunotherapy: T cell shielding through Nitric oxide/reactive oxygen species scavenging nanoparticles Potentiates anti-PD-1**. *Biomaterials* 2025, **315**:122904.

15.	Wang Z, Chen F, Cao Y, Zhang F, Sun L, Yang C, Xie X, Wu Z, Sun M, Ma F *et al*: **An Engineered Nanoplatform with Tropism Toward Irradiated Glioblastoma Augments Its Radioimmunotherapy Efficacy**. *Advanced materials (Deerfield Beach, Fla)* 2024, **36**(32):e2314197.

16.	Yan T, Liao Q, Chen Z, Xu Y, Zhu W, Hu P, Zhang S, Wu Y, Shu L, Liu J *et al*: **β-Ketoenamine covalent organic framework nanoplatform combined with immune checkpoint blockade via photodynamic immunotherapy inhibit glioblastoma progression**. *Bioactive materials* 2025, **44**:531-543.
