# Supplementary material for: Intranasal nano-immunotherapy for glioma: bypassing the blood–brain barrier, reprogramming the immune microenvironment, and bridging preclinical validation
Source: Front Immunol. 2026 Oct 20;17:1950362. doi: 10.3389/fimmu.2026.1950362 (PMC13728787; doi:10.3389/fimmu.2026.1950362)
Supplement: Supplementary file 3 [file Table3.docx]

**Table S3. Preclinical validation and advancement criteria for intranasal glioma nano-immunotherapy**

| **Validation domain** | **Essential assays/parameters** | **Recommended models** | **Key endpoints** | **Advancement criterion** | **PMID** |
| --- | --- | --- | --- | --- | --- |
| Nanoparticle characterization | Particle size, PDI, zeta potential, morphology, stability, encapsulation efficiency, loading capacity, release kinetics, and functional potency assay | Cell-free formulation testing; serum and nasal-fluid stability assays; independent production batches | Batch consistency, colloidal stability, payload retention, controlled release, and reproducible potency | Predefined critical quality attributes should remain within acceptance ranges across independent batches. | [1]; [2]; [3]; [4]; [5] |
| Manufacturing/CMC reproducibility | Source-material identity/purity, sterility/endotoxin, residual reagents, storage stability, scale-up performance, device compatibility, and delivered-dose uniformity | Independent production batches; pilot-scale/scale-up runs; clinically relevant nasal delivery-device testing | Lot-to-lot consistency, scalable yield, retained potency, release specifications, and product-device compatibility | A scalable GMP-compatible process should demonstrate reproducible composition and biological potency with predefined batch-release specifications. | [6]; [2]; [5] |
| Nasal formulation performance | Mucoadhesion, mucus penetration, viscosity, sprayability, epithelial permeability, enzymatic stability, and ciliary compatibility | Nasal epithelial cell models; ex vivo nasal mucosa; mucus diffusion/deposition assays | Nasal retention, epithelial transport, deposition reproducibility, and ciliary/epithelial compatibility | The formulation should show reproducible nasal performance without clinically meaningful epithelial or ciliary toxicity and should be compatible with a defined delivery device. | [1]; [2]; [7] |
| Cellular uptake and intracellular delivery | Uptake by glioma cells and relevant immune populations; intracellular trafficking; endosomal escape; cytosolic release | Glioma cells; microglia/macrophages; dendritic cells; endothelial cells; co-culture systems; orthotopic tumor tissue | Payload internalization, cytosolic availability, intended-cell uptake, and off-target uptake in normal brain cells | Reproducible uptake and intracellular delivery should be demonstrated in the intended cellular compartment with limited off-target uptake in normal brain. | [1]; [8]; [4]; [5] |
| Cell-specific target engagement | Cell-resolved siRNA knockdown, CRISPR editing, STING/TLR activation, CXCR4/CXCL12 blockade, or CD47/PD-L1 modulation | Target-cell cultures and immunocompetent orthotopic tumors with cell sorting, flow cytometry, or single-cell/spatial assays | Molecular target modulation and downstream pathway response in the intended glioma or immune-cell population | Target engagement should be demonstrated in the intended cell type rather than inferred from bulk tumor measurements alone. | [9]; [10]; [3]; [8]; [11]; [4]; [5] |
| Quantitative nose-to-brain biodistribution | Fluorescence/radiotracing, MRI/PET, tissue quantification, tumor-to-normal brain ratio, tumor-to-blood ratio, and systemic-organ distribution | Healthy animals and orthotopic glioma-bearing animals; repeated time-point sampling | Nasal retention, regional brain distribution, tumor accumulation, normal-brain exposure, and liver/spleen/kidney exposure | Quantitative and reproducible tumor enrichment relative to normal brain should be shown together with acceptable systemic exposure; whole-brain signal alone is insufficient. | [12]; [7]; [2]; [13]; [1] |
| Pharmacokinetics and temporal pharmacodynamics | Blood exposure, brain/tumor half-life, payload release, target-modulation duration, immune-response duration, and dose-response analysis | Repeated-dose animal studies with longitudinal sampling | Tumor exposure, systemic leakage, exposure-response relationship, duration of target engagement, and immune-response persistence | A tolerated dose should produce reproducible tumor exposure and mechanism-linked pharmacodynamics of sufficient duration to define an optimal biological dose. | [12]; [7]; [9]; [1]; [14] |
| Orthotopic efficacy | Bioluminescence/MRI, tumor burden, invasive-margin control, histology, and survival analysis | Immunocompetent orthotopic models (e.g., GL261 or CT-2A); additional patient-derived/humanized models when appropriate | Tumor control, invasive-margin response, median survival, and reproducibility across experiments | Advancement should require a reproducible survival benefit in an immunocompetent orthotopic glioma model rather than tumor shrinkage alone. | [9]; [1]; [12]; [7]; [15]; [3]; [16] |
| Spatial/state-resolved immune pharmacodynamics | Multiparametric flow cytometry, IHC/IF, scRNA-seq, spatial transcriptomics, multiplex imaging, cytokine profiling, and TCR sequencing | Immunocompetent orthotopic glioma models with longitudinal and spatial sampling | Tpex/transitional/Ttex composition; effector function; resident microglia versus monocyte-derived macrophage states; phagocytosis/antigen presentation; tumor-immune proximity; TCR clonality; persistence | Immune reprogramming should be cell-specific, functional, spatially relevant, and persistent; immune-cell abundance or cytokine changes alone are insufficient. | [9]; [15]; [11]; [8]; [17]; [10]; [3]; [16]; [18]; [5]; [19]; [20]; [14] |
| Combination therapy validation | Mechanism-based timing and sequencing with TMZ, radiotherapy, anti-PD-1/PD-L1, PDT, TMS, or ICD-inducing therapy | Orthotopic models receiving clinically relevant combination regimens | Synergy, survival extension, immune memory, recurrence delay, and combination toxicity | Combination benefit should be mechanism-driven and reproducible, with added efficacy not offset by disproportionate toxicity or unnecessary platform complexity. | [13]; [21]; [22]; [18]; [23]; [5] |
| Repeated-dose nasal/neurological/systemic safety | Nasal mucosa and olfactory epithelium histology, olfactory-bulb exposure, neuroinflammation, neurological/olfactory behavior, hematology, organ function, and cytokine release | Healthy and tumor-bearing animals; repeated-dose toxicity models | Mucosal injury, olfactory dysfunction, edema, microglial activation, neurological deficits, and systemic immune toxicity | No persistent mucosal/olfactory injury, progressive neuroinflammation, clinically meaningful neurological deficit, or major systemic toxicity should occur at the biologically active dose. | [9]; [1]; [10]; [3]; [4]; [5] |
| Survival, recurrence, and immune memory | Kaplan-Meier survival, long-term survivors, recurrence imaging, rechallenge, and post-treatment follow-up | Immunocompetent orthotopic glioma models | Median survival, durable response, recurrence control, and evidence of immune memory | Durable survival and recurrence control should support efficacy claims; transient tumor shrinkage alone should not justify translational advancement. | [12]; [7]; [13]; [3]; [18]; [5] |
| Imaging and biomarker bridging | MRI/radiomics, immune gene signatures, cytokines, CSF/plasma EVs, peripheral immune profiling, and route-specific safety biomarkers | Longitudinal preclinical imaging and biomarker studies aligned with early-phase trial design | Early response prediction, target/immune pharmacodynamics, pseudoprogression discrimination, and toxicity monitoring | A clinically feasible biomarker panel should distinguish delivery, target engagement, productive immune response, treatment effect, and toxicity during longitudinal monitoring. | [17] |

Note: Advancement criteria are intended to be evaluated collectively as practical go/no-go criteria. Failure in a critical domain - particularly tumor-selective delivery, cell-specific target engagement, survival benefit in an immunocompetent orthotopic model, repeated-dose safety, or manufacturing reproducibility - should trigger platform redesign rather than advancement based on antitumor activity alone. Abbreviations: CMC, chemistry, manufacturing, and controls; CRISPR, clustered regularly interspaced short palindromic repeats; CSF, cerebrospinal fluid; DC, dendritic cell; EV, extracellular vesicle; GMP, Good Manufacturing Practice; ICD, immunogenic cell death; IF, immunofluorescence; IHC, immunohistochemistry; MRI, magnetic resonance imaging; OMV, outer membrane vesicle; PDI, polydispersity index; PDT, photodynamic therapy; PET, positron emission tomography; scRNA-seq, single-cell RNA sequencing; TCR, T-cell receptor; TMZ, temozolomide; TMS, transcranial magnetic stimulation; Tpex, progenitor/stem-like exhausted T cells; Ttex, terminally exhausted T cells.

1.	Tang L, Zhang R, Wang Y, Zhang X, Yang Y, Zhao B, Yang L: **A simple self-assembly nanomicelle based on brain tumor-targeting peptide-mediated siRNA delivery for glioma immunotherapy via intranasal administration**. *Acta biomaterialia* 2023, **155**:521-537.

2.	Ferreira NN, Leite CM, Moreno NS, Miranda RR, Pincela Lins PM, Rodero CF, de Oliveira Junior E, Lima EM, Reis RM, Zucolotto V: **Nose-to-Brain Delivery of Biomimetic Nanoparticles for Glioblastoma Targeted Therapy**. *ACS applied materials & interfaces* 2025, **17**(1):484-499.

3.	Brüßeler MMT, Zam A, Moreno-Zafra VM, Rouatbi N, Hassuneh OWM, Marrocu A, Liam-Or R, Abdel-Bar HM, Walters AA, Al-Jamal KT: **Polyinosinic/Polycytidylic Lipid Nanoparticles Enhance Immune Cell Infiltration and Improve Survival in the Glioblastoma Mouse Model**. *Molecular pharmaceutics* 2024, **21**(12):6339-6352.

4.	Zou C, Liu X, Wang W, He L, Yin A, Cao Z, Zhu M, Wu Y, Liu X, Ma J *et al*: **Targeting GDF15 to enhance immunotherapy efficacy in glioblastoma through tumor microenvironment-responsive CRISPR-Cas9 nanoparticles**. *Journal of nanobiotechnology* 2025, **23**(1):126.

5.	You H, Zhang S, Zhang Y, Chen Q, Wu Y, Zhou Z, Zhao Z, Su B, Li X, Guo Y *et al*: **Engineered Bacterial Outer Membrane Vesicles-Based Doxorubicin and CD47-siRNA Co-Delivery Nanoplatform Overcomes Immune Resistance to Potentiate the Immunotherapy of Glioblastoma**. *Advanced materials (Deerfield Beach, Fla)* 2025, **37**(15):e2418053.

6.	Xu S, Yang H, Minev B, Ma W: **Biomimetic and personalized nanovaccines in cancer immunotherapy: Design innovations, translational challenges, and future directions**. *Journal of advanced research* 2026.

7.	Marrocco F, Falvo E, Mosca L, Tisci G, Arcovito A, Reccagni A, Limatola C, Bernardini R, Ceci P, D'Alessandro G *et al*: **Nose-to-brain selective drug delivery to glioma via ferritin-based nanovectors reduces tumor growth and improves survival rate**. *Cell death & disease* 2024, **15**(4):262.

8.	Hsieh HT, Huang HC, Chung CW, Chiang CC, Hsia T, Wu HF, Huang RL, Chiang CS, Wang J, Lu TT *et al*: **CXCR4-targeted nitric oxide nanoparticles deliver PD-L1 siRNA for immunotherapy against glioblastoma**. *Journal of controlled release : official journal of the Controlled Release Society* 2022, **352**:920-930.

9.	Mahajan AS, Dussold C, Kim S, Jarvis R, Hurley LA, Tommasini-Ghelfi S, Park J, Forsyth CM, Zhang B, Miska J *et al*: **cGAS-agonistic spherical nucleic acids reprogram the glioblastoma immune microenvironment and promote antitumor immunity**. *Proceedings of the National Academy of Sciences of the United States of America* 2025, **122**(45):e2409557122.

10.	Yu H, Liu W, Ding K, Wu J, Wang C, Wang S, Wu L, Tang Q, Yin X, Jiang K *et al*: **Sequential Release HydroLipo System for STING Gene Epigenetic Reprogramming and Immune Activation in Glioblastoma**. *Advanced science (Weinheim, Baden-Wurttemberg, Germany)* 2025, **12**(5):e2408323.

11.	Alghamri MS, Banerjee K, Mujeeb AA, Mauser A, Taher A, Thalla R, McClellan BL, Varela ML, Stamatovic SM, Martinez-Revollar G *et al*: **Systemic Delivery of an Adjuvant CXCR4-CXCL12 Signaling Inhibitor Encapsulated in Synthetic Protein Nanoparticles for Glioma Immunotherapy**. *ACS nano* 2022, **16**(6):8729-8750.

12.	Sandbhor P, Goda J, Mohanty B, Gera P, Yadav S, Chekuri G, Chaudhari P, Dutt S, Banerjee R: **Targeted nano-delivery of chemotherapy via intranasal route suppresses in vivo glioblastoma growth and prolongs survival in the intracranial mouse model**. *Drug delivery and translational research* 2023, **13**(2):608-626.

13.	Koh M, Lee JE, Ye E, Lee S, Kim HL, Song HE, Yoo HJ, Kim WJ, Park SM, Yang SH: **Intranasal administration of temozolomide conjugated nanoparticles with transcranial magnetic stimulation improves the survival in glioblastoma xenograft models**. *Drug delivery and translational research* 2026, **16**(2):509-521.

14.	Ma W, Sposito T, Wirtjes K, Jamieson C: **Persistent spatial immune responses in glioblastoma following oncolytic virus therapy**. *Cytokine & growth factor reviews* 2026, **89**:1-4.

15.	Wang T, Zhang H, Qiu W, Han Y, Liu H, Li Z: **Biomimetic nanoparticles directly remodel immunosuppressive microenvironment for boosting glioblastoma immunotherapy**. *Bioactive materials* 2022, **16**:418-432.

16.	Chen Y, Tian H, Zhang X, Nice EC, Huang C, Zhang H, Zheng S: **Copper-coordination driven brain-targeting nanoassembly for efficient glioblastoma multiforme immunotherapy by cuproptosis-mediated tumor immune microenvironment reprogramming**. *Journal of nanobiotechnology* 2024, **22**(1):801.

17.	Wei R, Li J, Lin W, Pang X, Yang H, Lai S, Wei X, Jiang X, Yuan Y, Yang R: **Nanoparticle-mediated blockade of CXCL12/CXCR4 signaling enhances glioblastoma immunotherapy: Monitoring early responses with MRI radiomics**. *Acta biomaterialia* 2024, **177**:414-430.

18.	Lee J, Kang Y, Lee H, Saravanakumar G, Park SA, Ahn S, Kim WJ: **Amplifying glioblastoma immunotherapy: T cell shielding through Nitric oxide/reactive oxygen species scavenging nanoparticles Potentiates anti-PD-1**. *Biomaterials* 2025, **315**:122904.

19.	Guo X, Ma S, Wang J, Fu Y, Ma W: **Terminally exhausted CD8(+) T cells in solid tumors: biology, biomarker potential and translational tools for precision oncology**. *Frontiers in immunology* 2025, **16**:1709852.

20.	Zhou W, Huang X, Jia G, Lu C, Ma W: **CD24 as an innate immune checkpoint in solid tumors: biology, biomarker stratification, and therapeutic translation**. *Frontiers in immunology* 2026, **17**:1798585.

21.	Tian T, Liang R, Erel-Akbaba G, Saad L, Obeid PJ, Gao J, Chiocca EA, Weissleder R, Tannous BA: **Immune Checkpoint Inhibition in GBM Primed with Radiation by Engineered Extracellular Vesicles**. *ACS nano* 2022, **16**(2):1940-1953.

22.	Wang Z, Chen F, Cao Y, Zhang F, Sun L, Yang C, Xie X, Wu Z, Sun M, Ma F *et al*: **An Engineered Nanoplatform with Tropism Toward Irradiated Glioblastoma Augments Its Radioimmunotherapy Efficacy**. *Advanced materials (Deerfield Beach, Fla)* 2024, **36**(32):e2314197.

23.	Yan T, Liao Q, Chen Z, Xu Y, Zhu W, Hu P, Zhang S, Wu Y, Shu L, Liu J *et al*: **β-Ketoenamine covalent organic framework nanoplatform combined with immune checkpoint blockade via photodynamic immunotherapy inhibit glioblastoma progression**. *Bioactive materials* 2025, **44**:531-543.
