# Supplementary material for: Intranasal nano-immunotherapy for glioma: bypassing the blood–brain barrier, reprogramming the immune microenvironment, and bridging preclinical validation
Source: Front Immunol. 2026 Oct 20;17:1950362. doi: 10.3389/fimmu.2026.1950362 (PMC13728787; doi:10.3389/fimmu.2026.1950362)
Supplement: Supplementary file 4 [file Table4.docx]

**Table S4. Clinical and translational evidence relevant to intranasal and nanoparticle-based glioma therapy**

| **Clinical evidence category** | **Agent/platform** | **Administration route** | **Trial stage/design** | **Human dosing/tolerability** | **Translational relevance** | **Major limitation** | **PMID / trial ID** |
| --- | --- | --- | --- | --- | --- | --- | --- |
| Human intranasal glioma therapy (non-nanoparticle) | Perillyl alcohol | Intranasal inhalation, four times daily | Phase I/II study in recurrent malignant glioma | 0.3% POH; 55 mg/dose (220 mg/day); tolerability and partial response/stable disease signals reported | Supports feasibility of repeated intranasal administration in glioma patients | Not a nanoformulation; not immune-directed; older non-randomized evidence | [1] |
| Human intranasal glioma therapy (non-nanoparticle) | Perillyl alcohol | Intranasal inhalation | Phase I/II clinical experience in recurrent GBM | 440 mg/day; survival signals reported versus historical/supportive-care controls | Supports feasibility of repeated intranasal dosing in recurrent GBM | Not nanoparticle-based; limited modern pharmacodynamic validation | [2] |
| Human intranasal glioma therapy (non-nanoparticle) | NEO100 (highly purified perillyl alcohol) | Intranasal nebulizer/nasal mask, four times daily | Open-label Phase I/IIa trial in recurrent GBM | 384, 576, 768, or 1152 mg/day in Phase I; no severe treatment-related adverse events; mild nasal symptoms reported | Provides modern human dosing and mucosal-tolerance data for repeated intranasal glioma therapy | Not nanocarrier-based or immune-payload-based; small Phase I cohort | [3]; NCT02704858 |
| Human glioma nanomedicine (non-intranasal) | AGuIX gadolinium-based theranostic nanoparticles | Intravenous, with radiotherapy and temozolomide | Phase I/II NANO-GBM trial | Clinical dose finding and MRI-based biodistribution; RP2D reported in early-phase data | Demonstrates clinical translation of nanoparticle-based radiosensitization in GBM | Not intranasal; not primarily designed for immune reprogramming | [4]; [5]; NCT04881032 |
| Human glioma nanomedicine (non-intranasal) | NanoTherm magnetic iron-oxide nanoparticles | Intratumoral/local placement plus alternating magnetic field | Clinical/registry experience in recurrent GBM | Clinical experience focused on tolerance and thermotherapy efficacy | Demonstrates that nanoparticle-based local therapy can reach clinical use in GBM | Not intranasal; not immune-payload-based; procedure-dependent | NCT06271421 |

**Note:** Clinical evidence categories distinguish direct human intranasal glioma therapy without a nanocarrier from human glioma nanomedicine using non-intranasal administration. These categories should not be interpreted as direct clinical evidence for intranasal nano-immunotherapy. PMID values are shown in EndNote temporary citation format; NCT identifiers are ClinicalTrials.gov registration numbers.

**Abbreviations:** GBM, glioblastoma; MRI, magnetic resonance imaging; POH, perillyl alcohol; RP2D, recommended Phase II dose.

1.	da Fonseca CO, Schwartsmann G, Fischer J, Nagel J, Futuro D, Quirico-Santos T, Gattass CR: **Preliminary results from a phase I/II study of perillyl alcohol intranasal administration in adults with recurrent malignant gliomas**. *Surgical neurology* 2008, **70**(3):259-266; discussion 266-257.

2.	da Fonseca CO, Simão M, Lins IR, Caetano RO, Futuro D, Quirico-Santos T: **Efficacy of monoterpene perillyl alcohol upon survival rate of patients with recurrent glioblastoma**. *Journal of cancer research and clinical oncology* 2011, **137**(2):287-293.

3.	Schönthal AH, Peereboom DM, Wagle N, Lai R, Mathew AJ, Hurth KM, Simmon VF, Howard SP, Taylor LP, Chow F *et al*: **Phase I trial of intranasal NEO100, highly purified perillyl alcohol, in adult patients with recurrent glioblastoma**. *Neuro-oncology advances* 2021, **3**(1):vdab005.

4.	Thivat E, Casile M, Moreau J, Molnar I, Dufort S, Seddik K, Le Duc G, De Beaumont O, Loeffler M, Durando X *et al*: **Phase I/II study testing the combination of AGuIX nanoparticles with radiochemotherapy and concomitant temozolomide in patients with newly diagnosed glioblastoma (NANO-GBM trial protocol)**. *BMC cancer* 2023, **23**(1):344.

5.	Biau J, Durando X, Boux F, Molnar I, Moreau J, Leyrat B, Guillemin F, Lavielle A, Cremillieux Y, Seddik K *et al*: **NANO-GBM trial of AGuIX nanoparticles with radiotherapy and temozolomide in the treatment of newly diagnosed Glioblastoma: Phase 1b outcomes and MRI-based biodistribution**. *Clinical and translational radiation oncology* 2024, **48**:100833.
